# A revision of the Larainae (Coleoptera, Elmidae) of Venezuela, with description of nine new species

**DOI:** 10.3897/zookeys.329.4961

**Published:** 2013-09-05

**Authors:** Crystal A. Maier

**Affiliations:** 1 Division of Entomology, Biodiversity Institute & Department of Ecology and Evolutionary Biology, University of Kansas, Lawrence, KS, 66045, USA

**Keywords:** Aquatic insects, Neotropical Region, riffle beetle, tepui, taxonomy

## Abstract

The species of the riffle beetle subfamily Larainae occurring in Venezuela are revised. Examination of 756 specimens yielded 22 species in nine genera occurring throughout the country. Seven species are newly recorded from the country: *Phanoceroides* sp. 1, *Phanocerus clavicornis* Sharp, 1882, *Phanocerus congener* Grouvelle, 1898, *Pharceonus volcanus* Spangler & Santiago-Fragoso, 1992, *Disersus dasycolus* Spangler & Santiago-Fragoso, 1992, *Disersus chibcha* Spangler & Santiago-Fragoso, 1987, and *Disersus inca* Spangler & Santiago-Fragoso, 1992. Nine species are found to be new to science, which are here described: *Hexanchorus dentitibialis* **sp. n.**, *H. falconensis* **sp. n.**, *H. flintorum* **sp. n.**, *H. homaeotarsoides* **sp. n.**, *H. inflatus* **sp. n.**, *Phanocerus rufus* **sp. n.**, *Pharceonus grandis* **sp. n.**, *Pharceonus ariasi* **sp. n.**, *Potamophilops bostrychophallus* **sp. n.** Additionally, a key to species, distribution maps, and photographs and genitalia illustrations are provided for all species.

## Resumen

Los escarabajos de la subfamilia Larainae de Venezuela son revisados. Se examinaron 756 especimenes, se reportan 22 especies en nueve géneros. Siete especies son nuevos registros para el pais – *Phanoceroides* sp. 1, *Phanocerus clavicornis* Sharp, 1882, *Phanocerus congener* Grouvelle, 1898, *Pharceonus volcanus* Spangler & Santiago-Fragoso, 1992, *Disersus dasycolus* Spangler & Santiago-Fragoso, 1992, *Disersus chibcha* Spangler & Santiago-Fragoso, 1987, y *Disersus inca* Spangler & Santiago-Fragoso, 1992. Se describen nueve especies nuevas – *Hexanchorus dentitibialis* **sp. n.**, *H. falconensis* **sp. n.**, *H. flintorum* **sp. n.**, *H. homaeotarsoides* **sp. n.**, *H. inflatus* **sp. n.**, *Phanocerus rufus* **sp. n.**, *Pharceonus grandis* **sp. n.**, *Pharceonus ariasi* **sp. n.**, *Potamophilops bostrychophallus* **sp. n.** Adicionalmente se presenta, una clave para las especies, mapas de distribución son presentadas. Fotografías e ilustraciones genitales se proporcionan para todas las especies.

## Introduction

The Elmidae, or “riffle beetles” are a widespread family of aquatic beetles common in a variety of running water habitats. Within Elmidae, there are two subfamilies, Larainae and Elminae. Species of Larainae are generally not found in the benthos, as with most elmine riffle beetles, but on water-splashed rocks and detritus just beyond the water’s edge, carrying with them an air bubble under a thin film of water. As adults, they are agile fliers and enter and exit the water with great ease (Kodada and Jäch 2005). These unique habitat requirements make laraines difficult to collect unless one is seeking them out specifically.

Currently, there are 27 genera of laraines described, with eleven of these known from the Neotropical Region. Most work on the Neotropical fauna has been relatively recent, with Spangler and Santiago-Fragoso (1987, 1992) revising the Central American and West Indian fauna and three genera. Three other genera have been described from the Guiana Shield in southern Venezuela, *Roraima* Kodada & Jäch, 1999 (Mount Roraima), and *Neblinagena* Spangler, 1985 and *Hypsilara* Maier & Spangler, 2011 (both from Cerro de Neblina). Additionally, new species of *Pharceonus* Spangler & Santiago-Fragoso, 1992 and *Potamophilops* Grouvelle, 1896 have been described from Ecuador and Brazil, respectively (Monte and Mascagni 2012; Fernandes and Hamada 2012).

Recent fieldwork to Venezuela has yielded a large volume of laraine specimens (756 individuals) that were unidentifiable to species using current literature. I assembled material from other collections, including types, and discovered several more new species in that material. Herein I describe all of the new species found on these and prior expeditions, record several new distribution records for Venezuela, and present a key to species of Larainae in Venezuela.

## Methods

Specimens were examined using an Olympus SZX7 dissecting microscope at various magnifications, from 8–56× magnification and described following terminology from Spangler and Santiago-Fragoso 1992. Specimens were photographed using a Canon EOS 70D with a Visionary Digital imaging system and photos were stacked using CombineZP image editing software (Hadley 2012).

The genitalia were extracted from relaxed specimens through the caudal opening in the abdomen. The genitalia were then cleared in heated potassium hydroxide for thirty minutes, rinsed with water, and temporarily mounted in glycerin gelatin (Zander 1997) for observation and illustration. The cleared genitalia were then placed in a plastic genitalia vial below the specimen for storage. Illustrations were made in pen and ink using a camera lucida attached to the compound microscope and scanned into the computer. Images were edited in Adobe® Illustrator® and Adobe® Photoshop®.

Label information in the material examined is quoted exactly from the original labels, with quotations (“…”) indicating breaks between labels and semicolons (;) indicating line breaks. Where label data are ambiguous or incorrect, interpretations of label data are given in brackets ([...]).

Specimens are deposited in the following collections:

CKB Jan Kodada Collection, Bratislava, Slovakia

FCC Fedor Čiampor Collection, Bratislava, Slovakia

MAIC Michael A. Ivie Collection, University of Montana, Bozeman, Montana, USA

MALUZ La Universidad del Zulia, Maracaibo, Venezuela

MIZA Museo del Instituto de Zoología Agrícola Maracay, Venezuela

NMPC National Museum, Prague, Czech Republic

NMW Naturhistorisches Museum, Vienna, Austria

SEMC Snow Entomological Collection, University of Kansas, Lawrence, Kansas, USA

USNM Smithsonian Institution, Washington, DC, USA

## Taxonomy

### Larainae species from Venezuela

*Disersus chibcha* Spangler & Santiago-Fragoso, 1987

*Disersus dasycolus* Spangler & Santiago, 1987

*Disersus inca* Spangler & Santiago, 1987

*Hexanchorus dentitibialis* sp. n.

*Hexanchorus falconensis* sp. n.

*Hexanchorus flintorum* sp. n.

*Hexanchorus homaeotarsoides* sp. n.

*Hexanchorus inflatus* sp. n.

*Hexanchorus mcdiarmidi* Spangler & Staines, 2003

*Hypsilara breweri* Ciampor et al., 2013

*Hypsilara royi* Maier and Spangler, 2011

*Neblinagena doylei* Kodada & Jäch, 1999

*Neblinagena prima* Spangler, 1985

*Phanoceroides* sp. 1

*Phanocerus clavicornis* Sharp, 1882

*Phanocerus congener* Grouvelle, 1898

*Phanocerus rufus* sp. n.

*Pharceonus ariasi* sp. n.

*Pharceonus grandis* sp. n.

*Pharceonus volcanus* Spangler & Santiago-Fragoso, 1992

*Potamophilops bostrychophallus* sp. n.

*Roraima carinata* Kodada & Jäch, 1999

#### Disersus

Sharp, 1882

http://species-id.net/wiki/Disersus

Figs 1, 2 , 9 –22

##### Diagnosis.

This genus can be distinguished from all other genera of Larainae that occur in South America by the following combination of characters: its large size (5.6–10.1 mm), distinct pronotum, which lacks a transverse depression across the apical third (Fig. 21), and elytron lacking an accessory stria (Fig. 18).

##### Distribution.

Members of this genus occur in Central and South America, as far north as Costa Rica and as far south as Cuzco, Peru (Spangler and Santiago-Fragoso 1987).

##### Habitat.

*Disersus* species can be found in fast-flowing streams and rivers, clinging to rocks and flying upstream in riffles and cascades. They rapidly enter and exit the water with great ease, carrying with them a silvery air bubble. They can be found in streams by looking for moving teardrop shaped air bubbles on the downstream sides of rocks with water cascading over them. Individuals of this genus are also commonly found at UV and mercury vapor lamps (pers. obs.).

**Figures 1–4.**
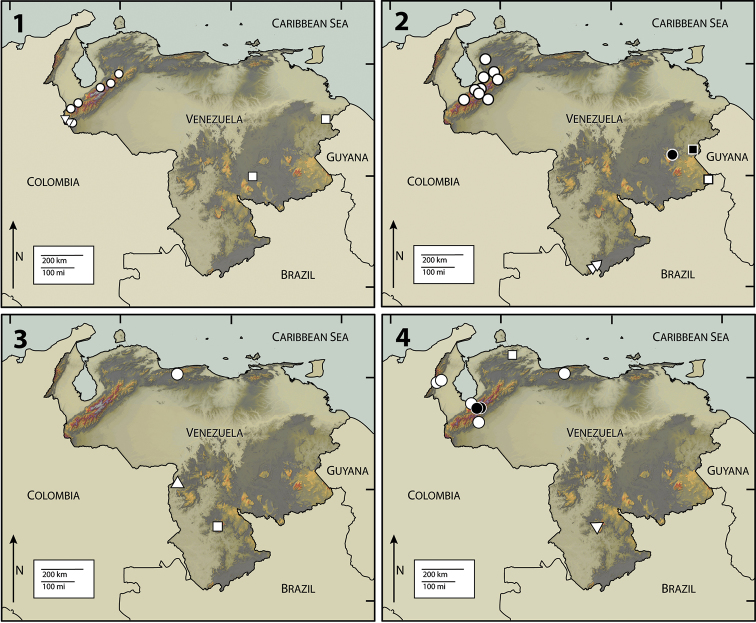
**1** Map of Venezuela, showing collection localities for *Disersus inca* (Triangle), *Disersus chibcha* (Circle), and *Potamophilops bostrychophallus* (Square) **2** *Disersus dasycolus* (White Circle), *Hypsilara royi* (White Triangle), *Roraima carinata* (White Square), *Hypsilara breweri* (Black square), *Hypsilara* sp. (Black Circle) **3** *Hexanchorus dentitibialis* (Circle), *Phanoceroides* sp. 1 (Triangle), *Hexanchorus homaeotarsoides* (Square) **4** *Hexanchorus falconensis* (White Square), *Hexanchorus mcdiarmidi* (White Circle), *Hexanchorus flintorum* (Black Circle), *Hexanchorus inflatus* (White Triangle).

**Figures 5–8.**
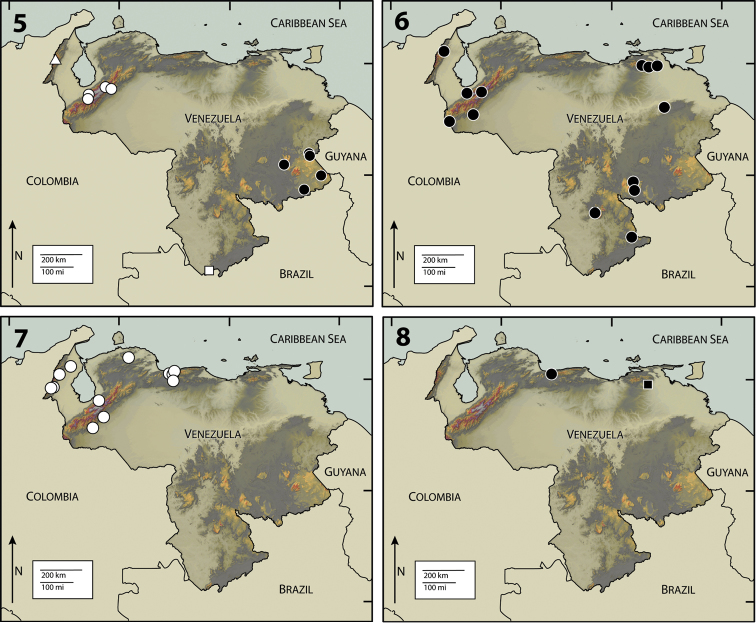
**5** Map of Venezuela, showing collection localities for *Pharceonus volcanus* (White Triangle), *Pharceonus grandis* (White Circle), *Neblinagena prima* (White Square), and *Neblinagena doylei* (Black Circle) **6** *Phanocerus clavicornis* (Black Circle) **7** *Phanocerus congener* (White Circle) **8** *Phanocerus rufus* (Black Circle), *Pharceonus ariasi* (Black Square).

**Figures 9–12.**
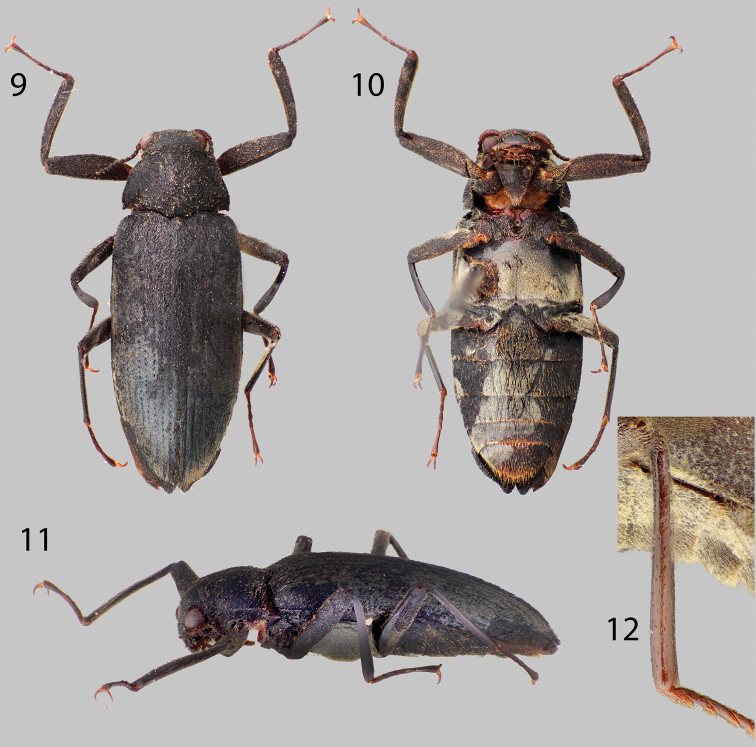
*Disersus chibcha*: **9** Dorsal habitus **10** Ventral habitus **11** Lateral habitus **12** Metatibia, male.

**Figures 13–17.**
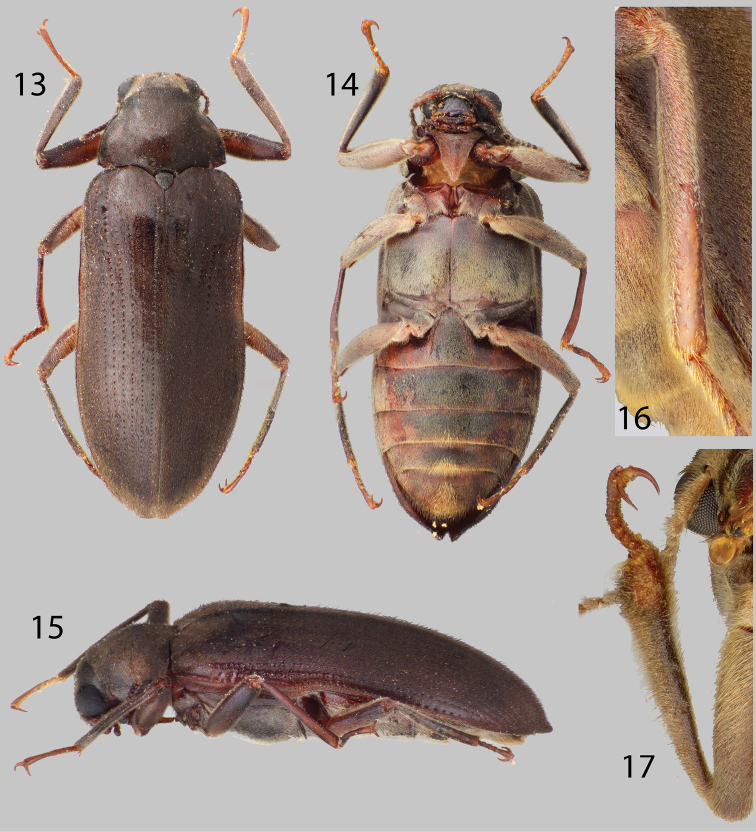
*Disersus dasycolus*: **13** Dorsal habitus **14** Ventral habitus **15** Lateral habitus **16** Metatibia, male **17** Protibia, male.

**Figures 18–22.**
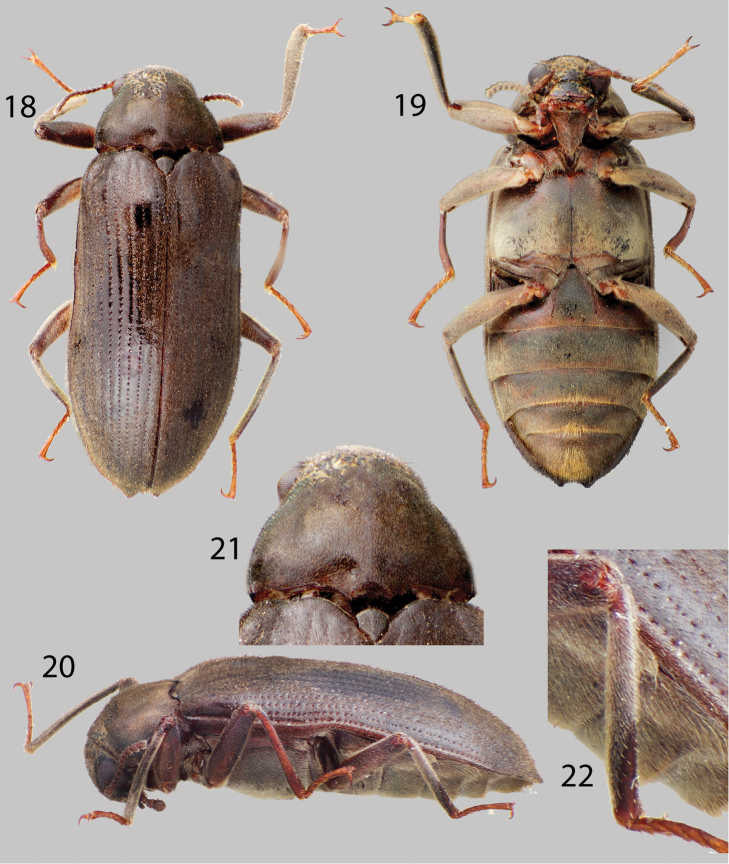
*Disersus inca*: **18** Dorsal habitus **19** Ventral habitus **20** Lateral habitus **21** Pronotum, dorsal view **22** Metatibia, male.

#### Disersus chibcha

Spangler & Santiago-Fragoso, 1987

http://species-id.net/wiki/Disersus_chibcha

Figs 1 , 9, 10, 11, 12

For complete description and genitalia illustrations see Spangler and Santiago-Fragoso 1987.

##### Material examined.

**COLOMBIA: Valle del Cauca:** “COLOMBIA: Dept Valle; 4km W. Cisneros; 28-II-69; R.E. Woodruff; Blacklight trap” (2 paratypes USNM). **VENEZUELA: Mérida State:** “VENEZUELA: Mérida State; 8°44.114'N, 71°26.898'W, 873m; ca. 2 km S La Azulita; Cascada la Palmita; 21.vii.2009; leg. Shepard; VZ09-0721-01Z” (1 specimen SEMC). “Venezuela Mérida; Dtto. Justo Briceño; Cañada de San Jose; 14/15-VII-1990” “Colector:; J. Camacho” (1 specimen MALUZ). **Táchira State:** “Rio Frio; Venezuela, Tachi-; ra [Táchira State]. 600m.; 2–10-IX-1981” “F. Fernandez. Y; J. Clavijo; A. Chacon” (5 specimens MIZA). “Rio Frio; Venezuela, Tachi-; ra [Táchira State]. 600m.; 20–24-IV-1982” “Expedicion; Zoologia; Agricola” (2 specimens MIZA). “Venezuela: Táchira; Quebrada La Uraca San; Felix 300m 17–22-vi-1998; J. DeMarmels y A. Chacon” (1 specimen MIZA). “Venezuela: Táchira; Quebrada La Uraca San; Felix 300m 7–16-v-2002; J. DeMarmels y A. Chacon” (2 specimens MIZA). “Venez. Táchira; Sn. Felix Rio Uraca; 350m; 12–19-iv-1996” “J. DeMarmels, A.; Chacon” (2 specimens MIZA). “Venezuela Táchira; La Blanca cr. Colon; La Fria 1000m; 14–21-iv-1998; Cols. J. DeMarmels; A. Chacon” (1 specimen MIZA). **Trujillo State:** “Venezuela Trujillo; Mcpio. Escuque; El Prado. Rio Buena Vista; 05-IV-1992” “J. Camacho; O. Lisano; Colectores” (2 specimens MALUZ).

##### Diagnosis.

This species of *Disersus* can be distinguished from its congeners by the combination of an almost entirely bare metatibia (Fig. 12); blackish-brown femora; and indistictly produced elytral apices (Fig. 9), as well as its large size (~8.5 mm) (see Spangler and Santiago-Fragoso 1987).

##### Intraspecific variation.

This species varies slightly in size and coloration, from dark brown to nearly black. Additionally, the last abdominal ventrite of the male is notched, while the last abdominal ventrite of the female is slightly sinuate, and the elytral apices of the female are more acute than those of the male (see Spangler and Santiago-Fragoso 1987).

##### Habitat and distribution.

Previously, *Disersus chibcha* was known only from a blacklight trap at Valle del Cauca, Colombia (Spangler and Santiago-Fragoso 1987). Subsequent collections of this species, given here, were from high gradient, medium-sized streams and waterfalls throughout the Mérida Andes in Venezuela (Fig. 1).

##### Associated species.

No other laraine species were collected with *Disersus chibcha*.

#### Disersus dasycolus

Spangler & Santiago-Fragoso, 1987

http://species-id.net/wiki/Disersus_dasycolus

Figs 2 , 13, 14, 15, 16, 17

For complete description and genitalia illustrations see Spangler and Santiago-Fragoso 1987.

##### Material examined.

**VENEZUELA: Mérida State:** “VENEZUELA: Mérida State; 8°35.355'N, 71°13.926'W 1646m; N. of Ejido, Rt. 4 river x-ing; 10.vii.2009; leg. Shepard; gross sample; VZ09-0720-02Z” (3 specimens SEMC). “VENEZUELA: Mérida State; 8°14.393'N, 71°48.672'W, 1862m; Bailadores; 18.vii.2009; Cascada; de Bailadores – stream margins; leg. Short et al.; VZ09-0718-02A” (1 specimen SEMC). “VENEZUELA: Mérida State; 8°38.006'N, 71°09.762'W, 2037m; Monte Zerpa area; 20.vii.2009; leg. W. Shepard; stream margin; gross sample; VZ09-0720-01Z” (3 specimens SEMC). “VENEZUELA: Mérida State; Rio Montealban, Rt. 4; 19 km. W. Mérida; 20 Feb 1976; C.M. & O.S. Flint Jr.” (2 specimens USNM). “Venezuela. Mérida; Mcipo. Rangel. P.N. Sierra; Nevada; La Mucuy; 2200m. 05/07-XI-1992” “Colector:; J. Camacho” (1 specimen MALUZ) “Via Jaji; Me. Vzla; 15-IX-77” “Cols:; J.M. Osario; A. Escalona” (1 specimen MALUZ). “La Pedregosa; Venezuela-Meri-; da. 1800m; 18-IX-1966” C.J. Rosales; J. Salcedo” (1 specimen MIZA). **Trujillo State:** “Bocono; Venezuela, Tru-; jillo. m.; 21-VII-1974” “F. Fernandez; H.M. Giani” (3 specimens MIZA). “Bocono; Trujillo Venz.; 13-VIII-1964” “E. Osuna; M. Gelbes” (1 specimen MIZA). “VENEZUELA: Trujillo State; 9°11.935'N, 70°45.233'W, 1601m; ca. 6 km E. Monte Carmelo; 22-vii-2009; leg. W. Shepard; VZ09-0722-03Z; gross sample” (1 specimen SEMC). “VENEZUELA, Trujillo; Parque N. Guaramacal; Mpio. Bocono, Laguna; Aguas Negras 1740; msnm. 26–28/V/1995” “Colectores:; J. Camacho; M. Garcia” (3 specimens MALUZ). “VENEZUELA: Trujillo; Paq. Nac. Guaramacal; Mpio. Bocono Laguna; de Aguas Negras, 1800; msnm. 20–25/VIII/ 1995; Trampa de Luz” “Colectores:; J. Camacho; M. Garcia” (2 specimens MALUZ). “VENEZUELA, Trujillo; Mcipo. Sta. Ana. Via; Bocono-Trujillo 1000; msnm. 28/V/1995” “Colectores:; Garcia, M.; Camacho, J.” (18 specimens MALUZ). **Lara State:** Anzoátegui; (Qda. Guago); Venezuela- Lara; 1440m 13–16-VI-72” “J. Salcedo; F. Zambrano” (10 specimens MIZA, 2 specimens MALUZ). **Barinas State:** “Venezuela: Barinas; San Isidro, 14 kms Sur; La Soledad. 1500m; 30–31-V-1975” “E.E. Dietz; leg.” (7 specimens MIZA).

##### Diagnosis.

*Disersus dasycolus* is unique among the species of *Disersus* and can be distinguished from all other species by the fuzzy protibiae of the male (Fig. 17). Males have a patch of dense, long, curly setae on the distal third of the ventral side of the protibia that is found in no other species of *Disersus* (Fig. 17) (See Spangler and Santiago-Fragoso 1987).

##### Intraspecific variation.

This species varies slightly in color and size, from a medium to dark brown. Females do not possess the patch of setae apicoventrally on the protibiae, and instead bear setae that resemble those of the other legs.

##### Habitat and distribution.

Previously, this species was only known from a single specimen collected at blacklight trap in Pastaza Province, Ecuador. Somewhat surprisingly, this species is quite common in streams in the Mérida Andes of Venezuela above 1000m elevation, and seems to prefer stream margins (Fig. 2).

##### Associated species.

A single species of *Hexanchorus*, *Hexanchorus flintorum* sp. n. was collected at the same localities as *Disersus dasycolus*. *Disersus* spp. are often collected in conjunction with *Hexanchorus* spp. as they seem to prefer the same rocky cascades as habitat. Other aquatic beetles collected at the same localities include: *Andogyrus* spp. (Gyrinidae).

#### Disersus inca

Spangler & Santiago-Fragoso, 1987

http://species-id.net/wiki/Disersus_inca

Figs 1 , 18, 19, 20, 21, 22

For complete description and genitalia illustrations, see Spangler and Santiago-Fragoso 1987.

##### Material examined.

**ECUADOR: Napo Province:** “ECUADOR. Napo; San Francisco; de Borja; 15 May 1975; at blacklight” “Collected by; Spangler, Gurney; Langley, & Cohen” (2 paratypes USNM). **VENEZUELA: Táchira State:** “Delicas; Venezuela – Tachi-; ra. 1500m; 27-IX-1966” “C.J. Rosales; J. Salcedo” (1 specimen MIZA). “VENEZUELA Táchira; La Pesa San Vincente de; La Revancha 2950m; 7–16-V-2002; J. DeMarmels, A. Chacon” (2 specimens MIZA). “VENEZUELA Táchira; La Provencia La; Revancha 1340m; 7–16-V-2002; J. DeMarmels, A. Chacon” (4 specimens MIZA).

##### Diagnosis.

*Disersus inca* can be distinguished from all other *Disersus* by the combination of the entirely pubescent metatibia, except for a narrow glabrous patch apically (Fig. 22) and the rounded elytral apices of the male.

##### Intraspecific variation.

Members of this species vary slightly in size and color, from a reddish-brown to medium brown, and in total length from 6.6–7.6 mm. The female differs from the male in having slightly produced elytral apices and a rounded apical abdominal ventrite (see Spangler and Santiago-Fragoso 1987).

##### Habitat and distribution.

Previously, *Disersus inca* was only collected in blacklight traps near montane streams in Ecuador, Colombia, and Peru (Spangler and Santiago-Fragoso 1987), this species is known to occur in the Cordillera Oriental in southwestern Venezuela from streams above 1000m in elevation (Fig. 1).

##### Associated species.

No other laraine species were collected with *Disersus inca*.

#### Hexanchorus

Sharp, 1882

http://species-id.net/wiki/Hexanchorus

Figs 3, 4 , 23 –49

##### Diagnosis.

*Hexanchorus* can be distinguished from all other Larainae genera that occur in South America by its small size (<5.1 mm) and the deep transverse groove across the apical third of the pronotum.

##### Distribution.

Species of *Hexanchorus* are widespread in the neotropics and can be found as far north as southern Mexico and as far south as Argentina. Additionally, a single species, *Hexanchorus caraibus* (Coquerel, 1851) is known from the West Indies south to Brazil (Spangler and Santiago-Fragoso 1992, Passos et al. 2009, Segura et al. 2012).

##### Habitat.

Often, *Hexanchorus* spp. can be found in the same habitats as *Disersus* spp. They are agile fliers and cling to rocks in fast flowing streams and rivers, flying into and out of the water quite readily.

##### Remarks.

Only a single species of *Hexanchorus* (*Hexanchorus mcdiarmidi*) was recorded from Venezuela prior to this study. Here I describe five new species from older museum material as well as specimens collected on recent expeditions to Venezuela.

**Figures 23–27.**
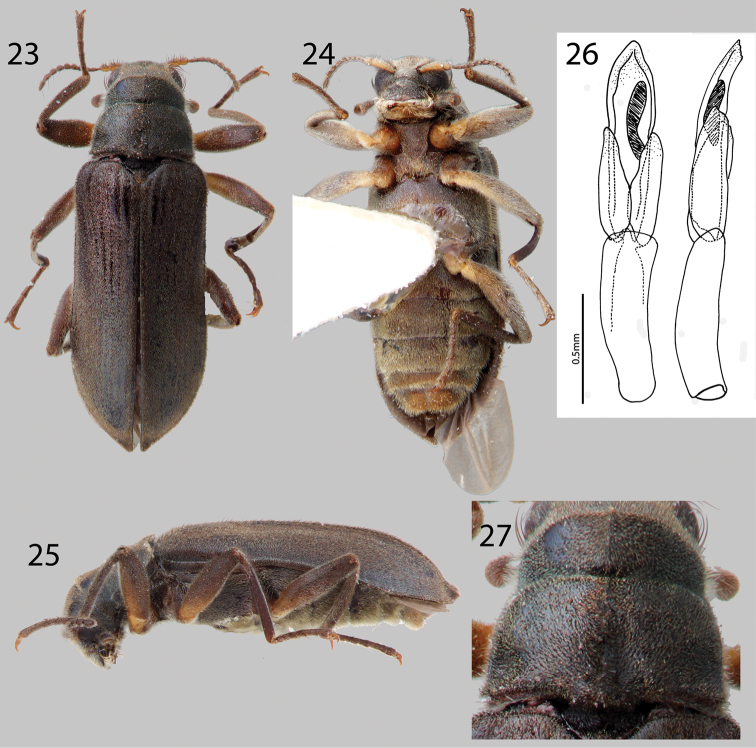
*Hexanchorus dentitibialis* sp. n.: **23** Dorsal habitus **24** Ventral habitus **25** Lateral habitus **26** Aedeagus, dorsal and lateral views **27** Pronotum, dorsal view.

**Figures 28–31.**
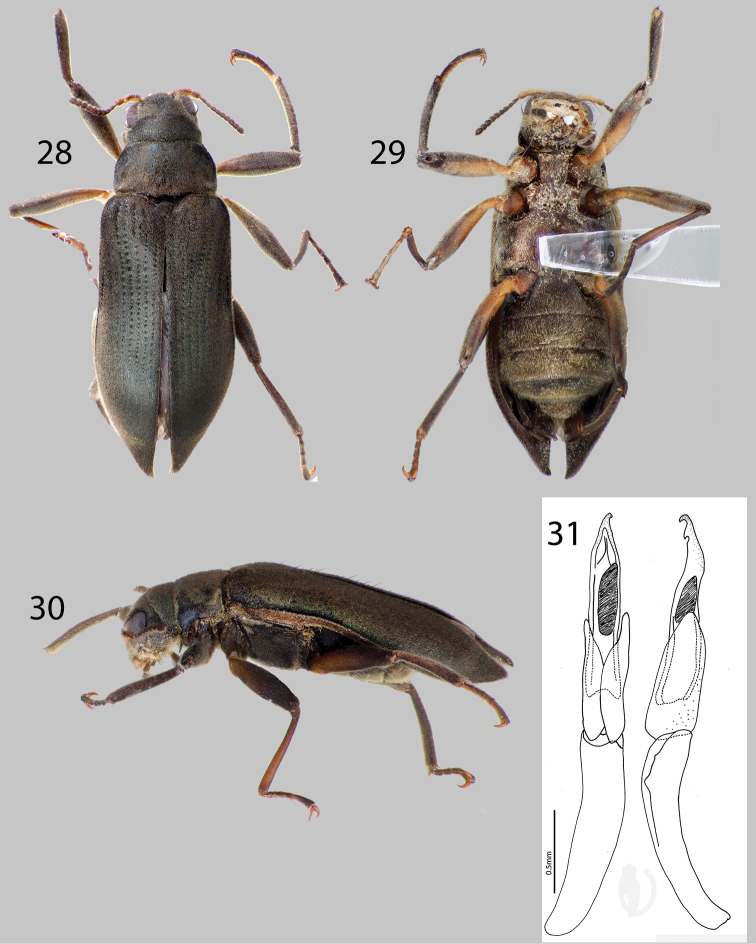
*Hexanchorus falconensis* sp. n.: **28** Dorsal habitus **29** Ventral habitus **30** Lateral habitus **31** Aedeagus, dorsal and lateral views.

**Figures 32–36.**
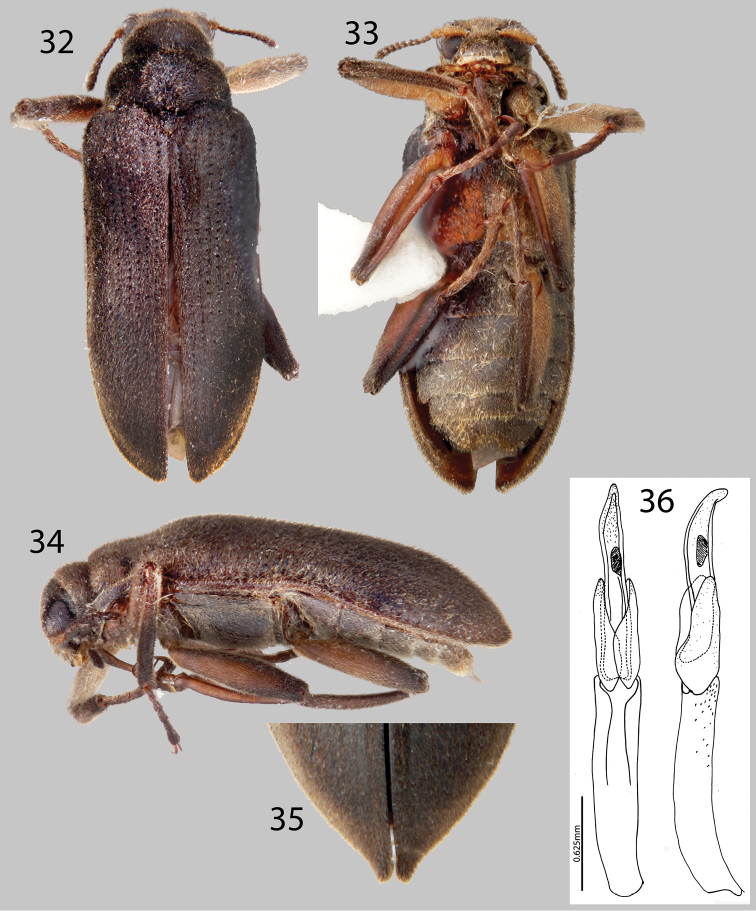
*Hexanchorus flintorum* sp. n.: **32** Dorsal habitus **33** Ventral habitus **34** Lateral habitus **35** Apices of elytra, female **36** Aedeagus, dorsal and lateral views.

**Figures 37–42.**
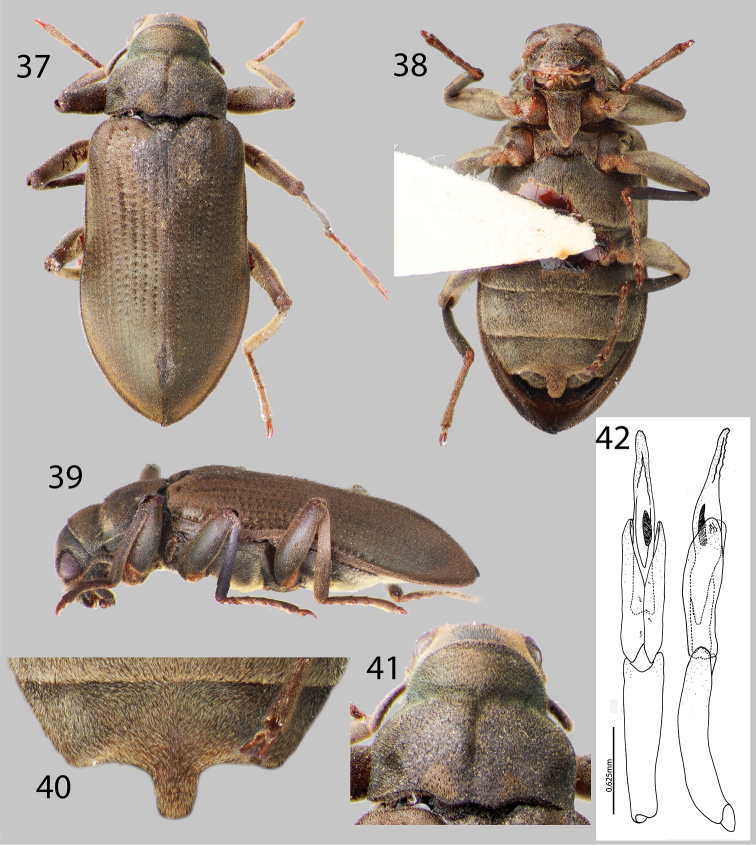
*Hexanchorus homaeotarsoides* sp. n.: **37** Dorsal habitus **38** Ventral habitus **39** Lateral habitus **40** Abominal ventrite 3, female **41** Pronotum, dorsal view **36** Aedeagus, dorsal and lateral views.

**Figures 43–47.**
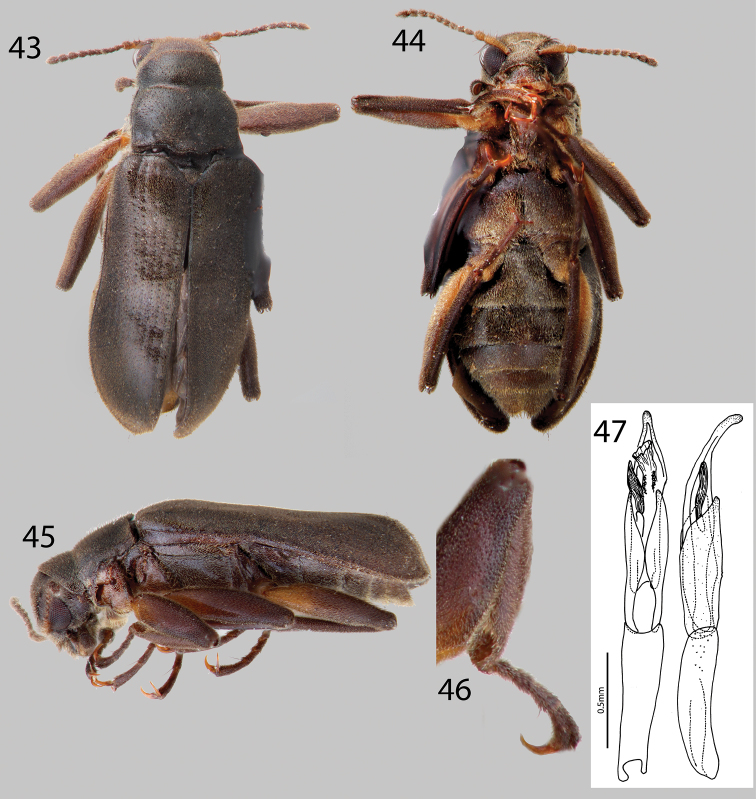
*Hexanchorus inflatus* sp. n.: **43** Dorsal habitus **44** Ventral habitus **45** Lateral habitus **46** Protibia, male, showing apical excavation **47** Aedeagus, dorsal and lateral views.

**Figures 48–49.**
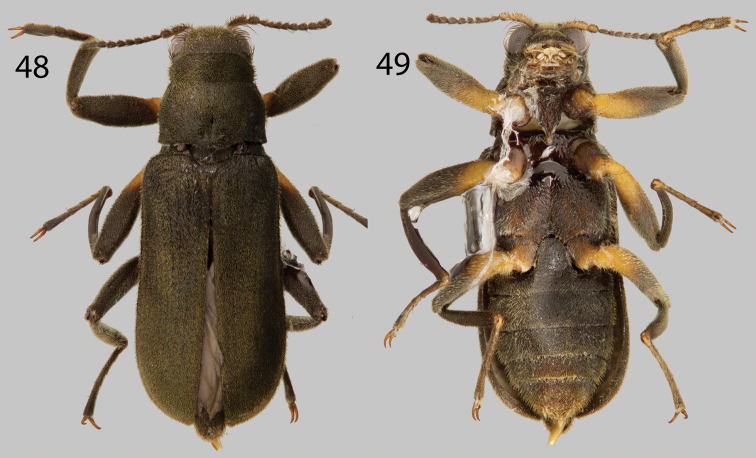
*Hexanchorus mcdiarmidi*: **48** Dorsal habitus **49** Ventral habitus.

#### Hexanchorus dentitibialis sp. n.

http://zoobank.org/675D8273-EE2C-4C81-B603-794E519445E9

http://species-id.net/wiki/Hexanchorus_dentitibialis

Figs 3 , 23, 24, 25, 26, 27

##### Type material.

**Holotype Male.**“Guayabita Cr.; Turmero: AR [Aragua State]; Venezuela; 466m 21-I-71; J. Salcedo; colr.” Holotype deposited in MIZA. **Paratypes (2):** Same locality data as holotype. Paratypes will be deposited in: 1 in SEMC, 1 in MIZA.

##### Diagnosis.

This species most closely resembles *Hexanchorus mcdiarmidi*, but can be distinguished from that species by the presence of a deep posteromedian impression on the pronotum (Fig. 27) and the extended elytral apices on the male (Fig. 23), which *Hexanchorus mcdiarmidi* lacks. Additionally, species identity can be confirmed by the distinctive aedeagus, which is truncate in lateral view (Fig. 26). This character does not occur in any other *Hexanchorus* species.

**Description.** Holotype Male. Body elongate, subparallel, dorsum moderately convex (Fig. 23). Length, 3.1 mm; greatest width, 0.9 mm. Body dark brown dorsally; venter dark brownish black. Base of antenna testaceous ventrally, and base of femur trochanter testaceous (Fig. 24). Dorsal surface densely covered with short recumbent setae; ventral surface densely covered with slightly longer, golden, recumbent setae (Fig. 23). Setae lacking greenish iridescence. Surface microreticulate, with dense fine punctures; fine punctures separated by distance equal to puncture diameter.

Head moderately coarsely, densely punctate; punctures separated by their diameter; cuticle microreticulate. Clypeus with anterior margin straight; angle on each side acute; lateral angles of clypeus with dense tuft of golden setae. Labrum with anterior margin entire and broadly rounded, covered with setae approximately twice as long as setae on head; lateral margins of clypeus with thick brush of curly, dark brown setae. Eyes hemispherical, narrowed posteriorly and bordered by long black curved setae (“eyelashes”) that arise near dorsal and ventral sides of eyes and extend toward middle of eye. Antenna pubescent, filiform; basal antennomeres I and II testaceous ventrally, with dense recumbent setae and long, dark brown setae (Fig. 23). Antennae without club, but thickening slightly towards apex. Antennae long, reaching past transverse groove of pronotum (Fig. 23). Apical antennomeres dark brownish black, with dense recumbent setae. Apical antennomere square.

Pronotum 0.8 mm long, 0.9 mm wide; with strong sublateral groove; lateral margins slightly sinuate; anterolateral angles square, not explanate; base of pronotum slightly sinuate and with broad lobe medially; posterolateral angles square, slightly explanate, declivous, depressed adjacent to each angle; discal area with fine, dense punctures, punctures separated by a distance equal to or less than their diameter; cuticle microreticulate (Fig. 27). Pronotal disc lacking median longitudinal depression, with slight postero-medial impression; disc covered with short, dense, recumbent setae (Fig. 27). Lateral projection of hypomeron truncate. Prosternum short in front of procoxae; lacking tuft of setae apicomedially. Prosternal process triangular, broad at base and tapering to apex; disc slightly impressed, with V-shaped groove; lateral margins reflexed; middle concave; apex acute (Fig. 24). Scutellum slightly longer than broad, flat, not distinctly elevated above the adjacent elytral intervals (Fig. 23). Mesoventrite short, depressed, with a deep, broad, V-shaped depression for reception of apex of prosternal process. Metaventrite with disc inflated on posterior three-fourths, finely punctate behind mesocoxae, punctures becoming more sparse laterally, with large, rounded depressions scattered on disc; with deep longitudinal groove on midline of disc, groove deepest and broadest on posterior third of disc; with short, dense pubescence; cuticular surface of metaventrite finely microreticulate.

Legs long and slender, dark brown (Fig. 24). Procoxae and metacoxae moderately widely separated; mesocoxae slightly more widely separated (Fig. 24). Protibia with deep excavation for reception of tarsi, with apical tooth (as in Fig. 46). Protarsus of male not expanded apicomedially (Fig. 24). Mesotibiae of male with medial pubescent area very long; lateral pubescent area only at extreme base (Fig. 23); with fine, short, nearly longitudinal carina on inner apex. Tarsal claws long and stout, light brown. Metafemur of male lacking internal glabrous patch.

Elytron with ten rows of fine punctures; punctures separated by a distance three to four times the diameter of the puncture; intervals with short, dense, recumbent pubescence and sparse erect setae; humeral area swollen (Fig. 23). Elytral striae visible apically. Elytron widening to about posterior two-thirds before converging to rounded apex. Apical third of elytron not inflated. Elytron without strong impression at basal third. Lateral bead of elytron slightly sinuate (Fig. 25). Elytra three times as long as pronotum; broadest point across humeri. Inner side of apex rounded; lateral margins smooth; humeri gibbous; elytral intervals slightly elevated; punctures on intervals no larger than finest punctures of head and pronotum and separated by 2–5 times puncture diameter.

Abdomen with five ventrites (Fig. 24). First three ventrites broadly, deeply depressed and distinctly carinate adjacent to metacoxae; carinae extending longitudinally behind metacoxae for almost entire length of first ventrite; cuticle densely covered with short, recumbent setae. Last ventrite shallowly and broadly emarginate (Fig. 24). Aedeagus wide, with truncate tip in lateral aspect, parameres reaching a little more than halfway up length of aedeagus (Fig. 26).

**Female.** Externally similar to male except elytral apices strongly pointed and slightly upturned (Fig. 23). Protibiae are slightly less curved than those of male. Mesotibiae without carina on inner apex. Metaventral disc not as deeply and less extensively concave. Abdominal sterna 1–3 convex, not concave.

##### Intraspecific variation.

This species varies slightly in color, length (3.1–3.3 mm), and degree of setation.

##### Etymology.

This species is named “dentitibialis”, meaning “toothed tibia” to describe the protibia, which has an excavation for reception of the tarsus and an apical tooth.

##### Distribution and habitat.

This species is only known from the type locality at Guayabita Creek, near Turmero, Aragua State, Venezuela (Fig. 3). The exact microhabitat is unknown.

##### Associated species.

No other Larainae species were collected with *Hexanchorus dentitibialis*.

#### Hexanchorus falconensis sp. n.

http://zoobank.org/4CA60E93-212C-4590-BC59-A692DC515A83

http://species-id.net/wiki/Hexanchorus_falconensis

Figs 4 , 28, 29, 30, 31

##### Type Material.

**Holotype Male.**“Venezuela: Falcón State; 11°10.667'N, 69°33.695'W 563 m; Cataratas del Hueque; scrubbing flat rocks in flow; leg. A. Short; VZ09-0709-01A” Holotype Deposited in MIZA. **Paratypes (9): VENEZUELA: Falcón State:** Same locality data as holotype (7 specimens SEMC). “Venezuela: Falcón State; 11°10.667'N, 69°33.695'W 563 m; Cataratas del Hueque; Short & Gustafson; leafpacks/roots; at river margin; VZ09-0709-01B” (1 specimen SEMC). “Venezuela: Falcón State; 11°10.667'N, 69°33.695'W 563 m; Cataratas del Hueque; leg. Shepard; mud puddles/pools; gross sample; VZ09-0709-01Z” (1 specimen SEMC). Paratypes will be deposited in: 1 in MALUZ, 1 in USNM, 7 in SEMC.

##### Diagnosis.

*Hexanchorus falconensis* can be distinguished from all other species of *Hexanchorus* by the distinctive aedeagus, with a “can-opener” tooth at apex (Fig. 31), and the extremely elongated elytral apices of the female (Fig. 28). Additionally, the pronotum lacks a median longitudinal impression (Fig. 28).

##### Description.

Holotype Male. Body elongate, subparallel, dorsum moderately convex. Length, 4.5 mm; greatest width, 1.1 mm. Body dark brown dorsally; venter dark brownish black (Fig. 28). Base of antenna testaceous ventrally, base of femur, trochanter testaceous (Fig. 29). Dorsal surface densely covered with short recumbent setae; ventral surface densely covered with longer, golden, recumbent setae. Setae with greenish iridescence (Fig. 28). Surface microreticulate, with dense fine punctures; fine punctures separated by distance equal to puncture diameter.

Head moderately coarsely, densely punctate; punctures separated by their diameter; cuticle microreticulate. Clypeus with anterior margin straight; angle on each side square; lateral angles of clypeus with dense turt of golden setae. Labrum with anterior margin entire and broadly rounded; labrum expanded laterally, covered with setae approximately three times as long as setae on head; lateral margins of clypeus with thick brush of curly, golden setae. Eyes hemispherical, narrowed posteriorly and bordered by long black curved setae (“eyelashes”) that arise near dorsal and ventral sides of eyes and extend toward middle of eye. Antenna pubescent, clubbed; basal antennomeres I and II testaceous ventrally, with dense recumbent setae and long, dark brown setae (Fig. 29). Antennae serrate, thickening slightly towards apex (Fig. 28). Antennae long, reaching past transverse groove of pronotum. Apical antennomeres dark brownish black, with dense recumbent setae. Apical antennomere pointed (Fig. 29).

Pronotum 1.0 mm long, 0.9 mm wide; with strong sublateral groove; lateral margins slightly sinuate; anterolateral angles square, not explanate; base of pronotum slightly sinuate and with broad lobe medially; posterolateral angles obtuse, slightly explanate, declivous, depressed adjacent to each angle; discal area with fine, dense punctures, punctures separated by a distance equal to or less than their diameter; cuticle microreticulate (Fig. 28). Pronotal disc lacking median longitudinal depression, with slight postero-medial impression; disc covered with short, dense, iridescent setae (Fig. 28). Lateral projection of hypomeron reduced, nearly absent. Prosternum short in front of procoxae; lacking tuft of setae apicomedially (Fig. 29). Prosternal process broadly triangular, broad at base and slightly tapering to apex; disc slightly impressed, without V-shaped groove; lateral margins reflexed; middle concave; apex broadly acuminate (Fig. 29). Scutellum slightly longer than broad, strongly convex, distinctly elevated above the adjacent elytral intervals. Mesoventrite short, depressed, with a deep, broad, V-shaped depression for reception of apex of prosternal process. Metaventrite with disc inflated on posterior three-fourths, finely punctate behind mesocoxae, punctures becoming more sparse laterally, with large, rounded depressions scattered on disc; with deep longitudinal groove on midline of disc, groove deepest and broadest on posterior third of disc; with short, dense pubescence; cuticular surface of metaventrite finely microreticulate.

Legs long and slender, dark brown (Fig. 29). Procoxae and metacoxae moderately widely separated; mesocoxae slightly more widely separated (Fig. 29). Protibia lacking excavation for reception of tarsi, lacking apical tooth. Protarsus of male strongly expanded apicomedially. Mesotibiae of male with medial pubescent area long; lateral pubescent area only at extreme base; with fine, short, nearly longitudinal carina on inner apex. Tarsal claws long and stout, light brown. Metafemur of male with small internal glabrous patch.

Elytron with ten rows of fine punctures; punctures separated by a distance three to four times the diameter of the puncture; intervals with short, dense, iridescent pubescence; humeral area swollen (Fig. 28). Elytral striae obscured apically. Elytron widening to about posterior two-thirds before converging to rounded and acute apex. Apical third of elytron not, or just barely inflated. Elytron without strong impression at basal third. Lateral bead of elytron sinuate. Elytra three times as long as pronotum; broadest point across humeri. Inner side of apex rounded; lateral margins smooth; humeri gibbous; elytral intervals slightly elevated; punctures on intervals no larger than finest punctures of head and pronotum and separated by 2–5 times puncture diameter.

Abdomen with five ventrites. First three ventrites broadly, deeply depressed and distinctly carinate adjacent to metacoxae; carinae extending longitudinally behind metacoxae for almost entire length of first ventrite; cuticle densely covered with short, recumbent setae. Last ventrite deeply and broadly emarginate. Aedeagus distinctive, with two small notches at tip (“can-opener” apex), parameres reaching more than halfway up length of aedeagus.

**Female.** Externally similar to male except elytral apices strongly acuminate and extended posteriorly (Fig. 28). Protibiae slightly less curved than those of male. Mesotibiae without carina on inner apex. Metaventral disc not as deeply and less extensively concave. Abdominal sterna 1–3 convex, not concave.

##### Intraspecific variation.

This species varies slightly in color, length (4.0–4.3 mm), and degree of setation.

##### Etymology.

This species is named “falconensis” in reference to the type locality in Falcón State, Venezuela.

##### Habitat and distribution.

*Hexanchorus falconensis* is only known from Cataratas del Hueque in Falcón State, Venezuela (Fig. 4). Specimens were collected in leaf packs and at stream margins, as well as in bulk samples and by scubbing rocks in the flowing water on flat, waterslide-like rocks.

##### Associated species.

This species was collected in association with *Phanocerus congener*. Other water beetles collected in the same habitat include: *Lutrochus vestitus* (Lutrochidae) (Maier & Short, 2013), *Heterelmis* spp., *Microcylloepus* spp., and *Onychelmis* spp. (Elmidae: Elminae), and the larvae of Psephenidae.

#### Hexanchorus flintorum sp. n.

http://zoobank.org/F6569AD8-7377-443F-B861-1210404C9BB3

http://species-id.net/wiki/Hexanchorus_flintorum

Figs 4 , 32, 33, 34, 35, 36

##### Type material.

**Holotype Male.**“VENEZUELA: Me. [Mérida State]; Rio Montealban, Rt. 4; 19km W. Mérida; 20 Feb. 1976; C.M. & O.S. Flint, Jr.” Holotype deposited in USNM. **Paratypes (22): VENEZUELA: Mérida State:** Same data as holotype (2 specimens USNM). “VENEZUELA: Mérida State; 8°35.355'N, 71°13.926'W, 1646m; N. of Ejido, Rt. 4 river x-ing; 10.vii.2009; leg. Shepard; gross sample; VZ09-0720-02Z” (20 specimens SEMC). Paratypes will be deposited in: 5 in USNM, 2 in MIZA, 1 in MALUZ, 14 in SEMC.

##### Diagnosis.

*Hexanchorus flintorum* can be distinguished from all other species of *Hexanchorus* that occur in Venezuela by the following combination of characters: pronotum without median longitudinal impression (Fig. 32), but with strong postero-median impression; parameres of male short, reaching less than half the length of the aedeagus (Fig. 36).

##### Description.

Holotype Male. Body elongate, subparallel, dorsum moderately convex. Length, 4.3 mm; greatest width, 1.5 mm. Body dark brown dorsally; venter dark brownish black (Fig. 32). Base of antenna, basal half of tibiae, and basal two thirds of femora light to medium brown (Fig. 33). Dorsal surface densely covered with short recumbent setae; ventral surface densely covered with short, recumbent setae and long, golden setae. Setae golden, and lacking greenish iridescence. Surface microreticulate, with dense fine punctures; fine punctures separated by distance equal to puncture diameter.

Head moderately coarsely, densely punctate; punctures separated by their diameter; cuticle microreticulate. Clypeus with anterior margin slightly concave; angle on each side acute. Labrum with anterior margin entire and gently concave; angle on each side acute, with row of dense, long golden setae. Eyes hemispherical, narrowed posteriorly and bordered by long black curved setae (“eyelashes”) that arise near dorsal and ventral sides of eyes and extend toward middle of eye. Antenna pubescent, clubbed; basal antennomeres I and II medium to light brown, with long setae, longer than width of segments, and dense recumbent setae; antennal club very loose, just slightly thickened towards apex (Fig. 32). Antennae short, reaching transverse groove of pronotum (Fig. 33). Apical antennomeres dark brownish black, with dense recumbent setae. Apical antennomere rounded.

Pronotum 1.1 mm long, 1.0 mm wide; with weak sublateral groove; lateral margins slightly sinuate; anterolateral angles obtuse, slightly explanate; base of pronotum slightly sinuate and with broad lobe medially; posterolateral angles obtuse, slightly explanate, declivous, depressed adjacent to each angle (Fig. 33); discal area with fine, dense punctures, punctures separated by a distance equal to or less than their diameter; cuticle microreticulate. Pronotal disc with slight median longitudinal drepression, with strong postero-medial impression; disc covered with golden setae. Lateral projection of hypomeron very acute, nearly absent. Prosternum short in front of procoxae; with tuft of dense golden setae apicomedially (Fig. 33). Prosternal process triangular, broad at base and tapering to apex; disc slightly impressed, without V-shaped groove; lateral margins reflexed; middle moderately longitudinally cariniform; apex narrow, acute (Fig. 33). Scutellum slightly longer than broad; flat, not at all convex, not distinctly elevated above the adjacent elytral intervals. Mesoventrite short, depressed, with a deep, broad, V-shaped depression for reception of apex of prosternal process. Metaventrite with disc inflated on posterior three-fourths, finely punctate behind mesocoxae, punctures becoming more sparse laterally, with large, rounded depressions scattered on disc; with deep longitudinal groove on midline of disc, groove deeper and broader on posterior third of disc (Fig. 33); with short, dense, short pubescence; cuticular surface of metaventrite finely microreticulate.

Legs long and slender, dark brown (Fig. 33). Procoxae and metacoxae moderately widely separated; mesocoxae slightly more widely separated (Fig. 33). Protibia only with very slight excavation for reception of tarsi, lacking apical tooth. Protarsus of male not expanded apicomedially. Mesotibiae of male with medial pubescent area long; lateral pubescent area only at extreme base; with fine, short, nearly longitudinal carina on inner apex (Fig. 33). Tarsal claws long and stout, light brown. Metafemur of male with internal glabrous patch.

Elytron with ten rows of fine punctures; punctures separated by a distance three to four times the diameter of the puncture; intervals with fine, golden, dense pubescence; humeral area swollen (Fig. 32). Elytral striae obscured apically. Elytron widening to about posterior two-thirds before converging to acute apex. Apical third of elytron not inflated. Elytron without strong impression at basal third. Lateral bead of elytron slightly sinuate. Elytra 3.5 times as long as pronotum; broadest point across humeri. Inner side of apex straight (Fig. 32); lateral margins smooth; humeri gibbous; elytral intervals slightly elevated; punctures on intervals no larger than finest punctures of head and pronotum and separated by 2–5 times puncture diameter.

Abdomen with five ventrites (Fig. 33). First three ventrites broadly, shallowly depressed and distinctly carinate adjacent to metacoxae; carinae extending longitudinally behind metacoxae for almost entire length of first ventrite; cuticle densely covered with long, golden setae (Fig. 33). Last ventrite deeply and broadly emarginate. Aedeagus strongly curved toward apex; parameres short, reaching less than half the length of the aedeagus (Fig. 36).

**Female.** Externally similar to male except inner apex of each elytron expanded posteriorly and slightly turned upward (Fig. 35). Protibiae slightly less curved than those of male. Metaventral disc not as deeply and less extensively concave. Abdominal sterna 1–3 convex, not concave.

##### Intraspecific variation.

This species varies slightly in color, length (3.9–4.2 mm, and degree of setation.

##### Etymology.

This species is named in honor of Dr. Oliver S. Flint and Mrs. Carol M. Flint who collected the specimens.

##### Distribution and habitat.

This species is only known from the mountains to the West of the Rio Chama valley near Ejido and Mérida, Mérida State, Venezuela, specifically, at Rio Montealban (Fig. 4). The largest series was collected from stream margins and pools at a river crossing north of Ejido.

##### Associated species.

The laraine elmid species *Disersus dasycolus* and *Pharceonus grandis* were collected in the same samples as *Hexanchorus flintorum*. Other aquatic beetles collected at the same localities include: *Andogyrus* spp. (Gyrinidae).

#### Hexanchorus homaeotarsoides sp. n.

http://zoobank.org/B1A05166-6309-4C12-A135-12033D6360BB

http://species-id.net/wiki/Hexanchorus_homaeotarsoides

Figs 3 , 37, 38, 39, 40, 41, 42

##### Type material.

**Holotype Male.**“Venezuela; Amazonas; P.N. Duida; Marahuaka” “Cabeceras del; Rio Yameduaca; 3°38'N, 65°28'W; 1–3-II-92, 1230m” “Exp. Terramar; J. Clavijo; A. Chacon” Holotype deposited in MIZA. **Paratypes (12): VENEZUELA: Amazonas State:** Same data as holotype (12 specimens MIZA). Paratypes will be deposited in: 7 in MIZA, 1 in MALUZ, 1 in USNM, 2 in SEMC.

##### Diagnosis.

*Hexanchorus homaeotarsoides* is distinctive in the genus, as it is one of only two species to possess a median projection on the third abdominal ventrite of the female. It differs from the only other species which bears this character, *Hexanchorus inflatus*, by its evenly rounded elytra and aedeagus with a saw tooth apex. Additionally, the antennae are short, not extending behind transverse impression of pronotum; and the pronotum has a strong median longitudinal impression and the apical third of the elytra are not inflated posteriorly.

##### Description.

Holotype Male. Body elongate, subparallel, dorsum moderately convex. Length, 4.2 mm; greatest width, 1.5 mm. Body dark brown dorsally (Fig. 37); venter dark brownish black (Fig. 38). Base of antenna light to medium brown. Dorsal surface densely covered with short recumbent setae; ventral surface densely covered with dense, short, recumbent setae. Setae lacking greenish iridescence (Fig. 38). Surface microreticulate, with dense fine punctures; fine punctures separated by distance equal to puncture diameter.

Head moderately coarsely, densely punctate; punctures separated by their diameter; cuticle microreticulate. Clypeus with anterior margin straight; angle on each side square. Labrum with anterior margin entire and gently rounded; angle on each side obtuse, covered with setae approximately twice as long as setae on head. Eyes hemispherical, narrowed posteriorly and bordered by long black curved setae (“eyelashes”) that arise near dorsal and ventral sides of eyes and extend toward middle of eye, setae not as prominent as in other species. Antenna pubescent, clubbed; basal antennomeres I and II medium to light brown, with dense recumbent setae and dense brushy light brown setae, lacking long setae (Fig. 39). Antennal club very loose, just slightly thickened towards apex. Antennae short, reaching transverse groove of pronotum. Apical antennomeres dark brownish black, with dense recumbent setae. Apical antennomere rounded.

Pronotum 1.1 mm long, 1.0 mm wide; with strong sublateral groove (Fig. 41); lateral margins slightly sinuate; anterolateral angles square, not explanate; base of pronotum slightly sinuate and with broad lobe medially; posterolateral angles obtuse, slightly explanate, declivous, depressed adjacent to each angle (Fig. 41); discal area with fine, dense punctures, punctures separated by a distance equal to or less than their diameter; cuticle microreticulate. Pronotal disc with strong median longitudinal depression, with strong postero-medial impression (Fig. 41); disc covered with short, dense setae. Lateral projection of hypomeron truncate. Prosternum short in front of procoxae; without tuft of dense golden setae apicomedially (Fig. 38). Prosternal process broadly triangular, broad at base and slightly tapering to apex; disc slightly impressed, without V-shaped groove; lateral margins reflexed; middle flattened; apex broadly, acuminate (Fig. 38). Scutellum slightly longer than broad, strongly convex, distinctly elevated above the adjacent elytral intervals. Mesoventrite short, depressed, with a deep, broad, V-shaped depression for reception of apex of prosternal process. Metaventrite with disc inflated on posterior three-fourths, finely punctate behind mesocoxae, punctures becoming more sparse laterally, with large, rounded depressions scattered on disc; with shallow longitudinal groove on midline of disc, groove deepest and broadest on posterior third of disc; with short, dense pubescence; cuticular surface of metaventrite finely microreticulate.

Legs long and slender, dark brown. Procoxae and metacoxae moderately widely separated; mesocoxae slightly more widely separated. Protibia lacking excavation for reception of tarsi, lacking apical tooth. Protarsus of male expanded apicomedially. Mesotibiae of male with medial pubescent area long; lateral pubescent area only at extreme base; with fine, short, nearly longitudinal carina on inner apex. Tarsal claws long and stout, light brown. Metafemur of male with internal glabrous patch.

Elytron with ten rows of fine punctures; punctures separated by a distance three to four times the diameter of the puncture; intervals with short, dense pubescence; humeral area swollen (Fig. 37). Elytral striae visible apically. Elytron widening to about posterior two-thirds before converging to rounded apex (Fig. 37). Apical third of elytron not, or just barely inflated. Elytron without strong impression at basal third. Lateral bead of elytron strongly sinuate. Elytra 3.5 times as long as pronotum; broadest point across humeri. Inner side of apex angled; lateral margins smooth; humeri gibbous; elytral intervals slightly elevated; punctures on intervals no larger than finest punctures of head and pronotum and separated by 2–5 times puncture diameter.

Abdomen with five ventrites. First three ventrites broadly, shallowly depressed and distinctly carinate adjacent to metacoxae; carinae extending longitudinally behind metacoxae for almost entire length of first ventrite; cuticle densely covered with short, recumbent setae. Last ventrite deeply and broadly emarginate. Aedeagus unique in configuration, acute, with row of small teeth at apex; parameres long, extending past halfway up aedeagus (Fig. 42).

**Female.** Externally similar to male, except inner apex of each elytron acute and slightly turned upward (Fig. 37). Protibiae slightly less curved than those of male. Mesotibiae without carina on inner apex. Metaventral disc not as deeply and less extensively concave. Abdominal sterna 1–3 convex, not concave; apicomedial margin of third ventrite with distinct posterior projection (Fig. 40).

##### Intraspecific variation.

This species varies slightly in color, length (4.1–5.1 mm), and degree of setation.

##### Etymology.

The specific epithet, “homaeotarsoides” is a reference to the third ventrite of the female, which bears resemblance to the abdominal ventrite 4 of the male in the rove beetle (Staphylinidae) genus *Homaeotarsus*.

##### Distribution and habitat.

This species is known only from the type locality at Rio Yameduaca in Amazonas State, Venezuela (Fig. 3). Nothing is known about its habits and exact microhabitat preferences.

##### Associated species.

*Hexanchorus inflatus* sp. n.occurs in the same region of Venezuela, but no other species of laraine elmid has been collected at this locality.

#### Hexanchorus inflatus sp. n.

http://zoobank.org/3C6A344A-9613-43E6-A4D6-7DD05204A98A

http://species-id.net/wiki/Hexanchorus_inflatus

Figs 4 , 43, 44, 45, 46, 47

##### Type material.

**Holotype Male.**“Venezuela Exp[edition].; Territ. Amazonas; Upper Cunucunuma; Tapara Apr. 20, 1950” “J. Maldonado; Capriles Coll.” Holotype deposited in USNM. **Paratypes (5): VENEZUELA: Amazonas State:** Same data as holotype (5 Specimens). Paratypes will be deposited in: 4 in USNM, 1 in SEMC.

##### Diagnosis.

This species can be distinguished from all other *Hexanchorus* species that occur in Venezuela by the following combination of characters: antennae long (Fig. 44), extending behind transverse impression of pronotum; pronotum lacking median longitudinal impression (Fig. 43); and protibia with deep excavation apically (Fig. 46). It most closely resembles *Hexanchorus homaeotarsoides* in distribution and that the female bears a median projection of the third ventrite, but can be distinguished from the former species by the distinctly inflated elytral apices (Fig. 45), and aedeagus with a smooth apex (Fig. 47).

##### Description.

Holotype Male. Body elongate, subparallel, dorsum moderately convex (Fig. 43). Length, 2.5 mm; greatest width, 1.1 mm. Body dark brown dorsally; venter dark brownish black (Fig. 44). Base of antenna and base of femora light to medium brown. Dorsal and ventral surface densely covered with short recumbent setae (Figs 43 and 44). Setae golden, and lacking greenish iridescence. Surface microreticulate, with dense fine punctures; fine punctures separated by distance equal to puncture diameter and coarse punctures confluent to separated by 1–3 times puncture diameter.

Head moderately coarsely, densely punctate; punctures separated by their diameter; cuticle microreticulate. Clypeus with anterior margin truncate; angle on each side broadly rounded. Labrum with anterior margin entire and gently arcuate; angle on each side broadly rounded, with row of dense, long golden setae. Eyes hemispherical, narrowed posteriorly and bordered by long black curved setae (“eyelashes”) that arise near dorsal and ventral sides of eyes and extend toward middle of eye. Antenna pubescent, filiform, nearly serrate (Fig. 44); basal antennomeres I and II medium to light brown, with long setae, longer than width of segments, and dense recumbent setae; antenna lacking club. Antennae long, reaching past transverse groove of pronotum. Apical antennomeres dark brownish black, with dense recumbent setae (Fig. 44). Apical antennomere rounded.

Pronotum 1.2 mm long, 1.1 mm wide; lacking sublateral groove (Fig. 43); lateral margins slightly sinuate; anterolateral angles square, not explanate or depressed; base of pronotum slightly sinuate and with broad lobe medially; posterolateral angles obtuse, slightly explanate, declivous, depressed adjacent to each angle; discal area with fine, dense punctures, punctures separated by a distance equal to or less than their diameter; cuticle microreticulate. Pronotal disc lacking median longitudinal depression, with weak postero-medial impression; disc with sparse, short setae (Fig. 43). Lateral projection of hypomeron acute. Prosternum short in front of procoxae; lacking tuft of setae and dense golden setae apicomedially (Fig. 44). Prosternal process triangular, broad at base and tapering to apex; disc with V-shaped groove; lateral margins reflexed; middle moderately longitudinally cariniform; apex narrow, acute (Fig. 44). Scutellum slightly longer than broad; very strongly convex, distinctly elevated above the adjacent elytral intervals. Mesoventrite short, depressed, with a deep, broad, V-shaped depression for reception of apex of prosternal process. Metaventrite with disc inflated on posterior three-fourths, finely punctate behind mesocoxae, punctures becoming more sparse laterally, with large, rounded depressions scattered on disc; with shallow longitudinal groove on midline of disc, groove deepest and broadest on posterior third of disc; with dense, short pubescence; cuticular surface of metaventrite finely microreticulate (Fig. 44).

Legs long and slender, dark brown (Fig. 45). Procoxae and metacoxae moderately widely separated; mesocoxae slightly more widely separated. Protibia with apical rounded excavation for reception of tarsi, with apical tooth (Fig. 46). Protarsus of male expanded apicomedially. Mesotibiae of male with medial pubescent area long; lateral pubescent area only at extreme base; with fine, short, nearly longitudinal carina on inner apex. Tarsal claws long and stout, light brown. Metafemur of male with internal glabrous patch.

Elytron with ten rows of fine punctures; punctures separated by a distance three to four times the diameter of the puncture; intervals with fine, short, dense pubescence; humeral area moderately swollen (Fig. 43). Elytral striae obscured apically. Elytron widening to about posterior two-thirds before converging to strongly rounded apex (Fig. 43). Apical third of elytron strongly inflated, most evident in lateral view (Fig. 45). Elytron with strong impression at basal third. Lateral bead of elytron straight. Elytra three times as long as pronotum; broadest point across humeri but only slightly broader than broadest point at apical third; inner side of apex rounded; lateral margins smooth; humeri gibbous; elytral intervals flat; punctures on intervals no larger than finest punctures of head and pronotum and separated by 2–5 times puncture diameter.

Abdomen with five ventrites (Fig. 44). First three ventrites broadly, shallowly depressed and distinctly carinate adjacent to metacoxae; carinae extending longitudinally behind metacoxae for almost entire length of first ventrite; cuticle densely covered with setae. Last ventrite deeply and broadly emarginate (Fig. 44). Aedeagus broadly curved, with smooth apex, parameres long, reaching more than halfway up length of aedeagus (Fig. 47).

**Female.** Externally similar to male except inner apex of each elytron acute and slightly turned upward. Protibiae slightly less curved than those of male. Mesotibiae without carina on inner apex. Metaventral disc not as deeply and less extensively concave. Abdominal sterna 1–3 convex, not concave; apicomedial margin of third ventrite with distinct posterior projection.

##### Intraspecific variation.

This species varies slightly from black to dark brown in color. There are differences in size (2.5– 2.9 mm TL) and slight differences in punctation and setation.

##### Etymology.

This species is named “inflatus” for the distinct elytra, which appear inflated posteriorly.

##### Habitat and distribution.

This species is only known from specimens collected on an expedition by the University of Puerto Rico in 1950. They were collected at one locality from the upper Rio Cunucunuma in Amazonas State, Venezuela, north of Cerro Duida (Drake and Capriles 1952) (Fig. 4). The habits and microhabitat preferences of this species are unknown.

##### Associated species.

*Hexanchorus homaeotarsoides* sp. n. occurs in the same region of Venezuela, but no other species of laraine elmid has been collected at this locality.

#### Hexanchorus mcdiarmidi

Spangler & Staines, 2003

http://species-id.net/wiki/Hexanchorus_mcdiarmidi

Figs 4 , 48, 49

For a complete description and illustrations of genitalia, see Spangler and Staines 2003.

##### Material examined.

**VENEZUELA: Barinas State:** “VENEZUELA: Barinas State; 8°03.341'N, 70°56.597'W, 203m; nr. Quiri, Rio Quiu; 15.vii.2009; leg. W. Shepard; ex. bulk sample; VZ09-0715-03Z” (99 specimens SEMC). **Distrito Capital:**“VENEZUELA, Dto. Federal; Los Caracas; 19 January 1985; P. Spangler, R. Faitoute; W. Steiner, & A. Conover” (6 paratypes USNM) **Mérida State:** “VENEZUELA: Mérida State; 8°57.205'N, 71°17.620'W, 88m; W. of Tucani; 21.vii2009; Gross sample from river; leg. Shepard; VZ09-0721-04Z” (17 specimens SEMC). **Zulia State:** “VENEZUELA: Zulia State; 9°50.513'N, 72°48.334'W. 252m; Perija N.P. Tukuko: Rio Tukuko; 5.vii.2009; leg. Short & Gustafson; riffle/rocks in river; VZ09-0705-01B” (96 specimens SEMC). “VENEZUELA: Zulia State; P.N. Perija: Tukuko; 18 vii.2008; Rio Marpito” “Andrew E. Z.; Short, leg.” (5 specimens SEMC). “VENEZUELA: Zulia El; Tucuco (51 km S.O. de; Machiques); 24-VI-1992” “Colector:; J. Camacho” (11 specimens MALUZ).

##### Diagnosis.

*Hexanchorus mcdiarmidi* can be disinguished from all other *Hexanchorus* species in Venezuela by the presence of a greenish iridescent sheen on the dorsal setae (Fig. 48), the narrow parameres, and pronotum with only a shallow postero-median impression (Fig. 48).

##### Intraspecific variation.

This species varies slightly from black to dark brown in color, additionally, there may be slight variation in the greenish iridescence of the elytral setae. *Hexanchorus mcdiarmidi* has a purplish and orange iridescent sheen while submerged in alcohol (Short, pers. comm.). There is variation in size (2.9–3.4 mm TL) and slight differences in punctation among specimens.

##### Habitat and distribution.

*Hexanchorus mcdiarmidi* is widespread in lower altitude (<300 m elevation) streams throughout western Venezuela (Fig. 4). They can be found in dense aggregations on water-splashed emergent rocks in small to medium sized swift-flowing streams and rivers with rocky substrate.

##### Associated species.

While no other species of *Hexanchorus* were collected at the same localities as *Hexanchorus mcdiarmidi*, other laraines collected in the same habitats include *Disersus dasycolus*, *Phanocerus congener* and *Phanocerus clavicornis*, and *Pharceonus volcanus*. Other water beetles collected at the same sites include: *Anacaena* spp. and *Enochrus* spp. (Hydrophilidae).

#### Hypsilara

Maier & Spangler, 2011

http://species-id.net/wiki/Hypsilara

**Figures 50–53.**
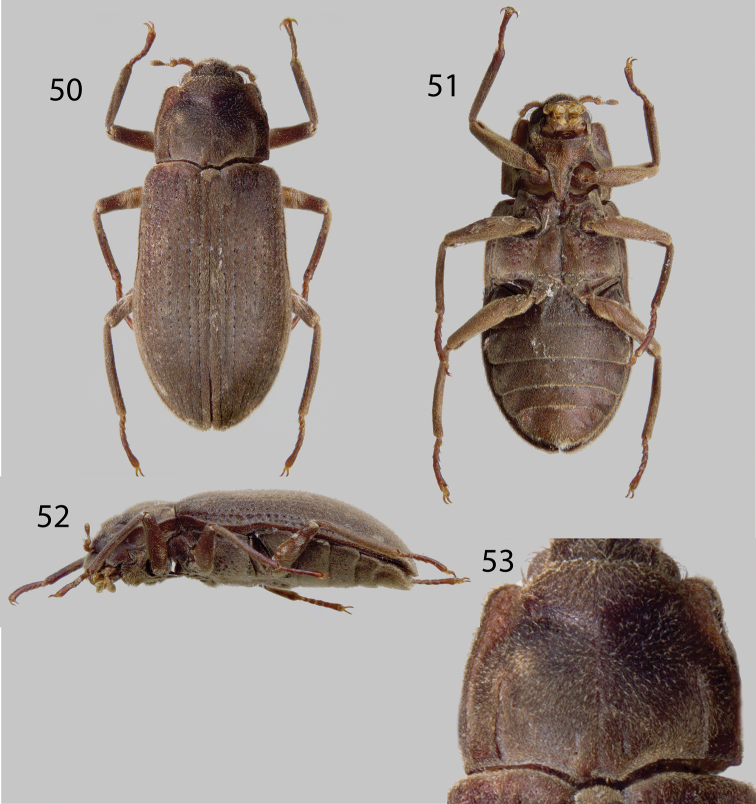
*Hypsilara royi*: **50** Dorsal habitus **51** Ventral habitus **52** Lateral habitus **53** Pronotum, dorsal view. (Modified from Maier and Spangler 2011).

Figs 2 , 50, 51, 52, 53

##### Diagnosis.

This genus can be distinguished from all other laraine genera by its small size (ca. 4.5 mm), and the presence of a shallow, wide, V-shaped groove across apical third of the pronotum, which lacks strong gibbosities or protuberances (Fig. 53).

##### Distribution.

Currently, *Hypsilara* is only known from near the base of Cerro de Neblina, Amazonas State and Gran Sabana, Venezuela (Fig. 2).

##### Habitat.

From Maier and Spangler 2011 “These are known a small, shallow brook about one to two meters wide and with occasional pools about one meter deep, with a substratum of sand, boulders, and bedrock. This small tributary originates on Cerro de la Neblina and feeds the Rio Baria, which drains most of the massif. The high water marks and polished boulders along the stream bed indicate that in times of heavy rainfall, the brook becomes scoured by flash flooding. Paratypes were collected from similar small streams at high elevations.

Water quality data obtained by using colorimetric analyses of the brook at the type-locality are as follows; pH: 4, hardness: 0, oxygen: 9 ppm. The air temperature was 21°C and the water temperature was 17°C when the analyses were made.”

#### Hypsilara breweri

Čiampor et al., 2013

http://species-id.net/wiki/Hypsilara_breweri

Fig. 2 (See figures 1–9 in Čiampor et al. 2013) 

For complete species descriptions and genitalia illustrations, see Čiampor et al. 2013.

##### Diagnosis.

This species can be distinguished from all other species of *Hypsilara* by the unique adeagus, possessing the following characters: long phallobase (ca. 0.6x as long as median lobe) and short parameres (ca. 0.67x as long as median lobe) (Čiampor et al. 2013).

##### Distribution and habitat.

This species is known only from the type locality at a small, tannic stream flowing in degraded forest at Sierra de Lema, Gran Sabana, Venezuela (Fig. 2). The species was collected from submerged woody debris, roots, and leaf litter (Čiampor et al. 2013)

##### Notes.

This species was described in a recent paper by Čiampor et al. (2013) and represents the second species described in the genus *Hypsilara*. A third species was also discovered at Auyán tepui, but not named, as it is known only from a single female (Fig. 2). Additionally, based on analysis of the *cox* 1 gene, they found that this genus is likely to be closely related to *Phanoceroides*, an unusual laraine of north and central South America (Čiampor et al. 2013).

#### Hypsilara royi

Maier & Spangler, 2011

http://species-id.net/wiki/Hypsilara_royi

Figs 2 , 50, 51, 52, 53

See Maier and Spangler 2011 for complete description and genitalia illustrations.

##### Type material examined.

“VENEZUELA: T. F. AMAZ. [Amazonas State]; Cerro de la Neblina; Camp XI 1450 m; 00°52'N, 65°58'W” “at stream; 26–27 February 5 l985; P. J. and P. M. Spangler, R. A. Faitoute; collector”. (1 Holotype MIZA, 15 Paratypes). “VENEZUELA: T. F. AMAZ.; Cerro de la Neblina; Camp X, 00°54'N, 60°2'W, 1690m, 12–13 February l985, W. Steiner” (16 paratypes). “VENEZ., T.F.A. [Amazonas State];C.d.l. [Cerro de la] Neblina; Base camp; 26–31 Jan. 1985; Flite [sic] intercept Pan; Trap” (1 paratype). “VENEZUELA: T. F. AMAZ. [Amazonas State]; Cerro de la Neblina; Camp X, 1690m; 00°54'N, 60°2'W; 12 February l985” “Small sunlit stream; leaf packs in falls; between boulders; W. E. Steiner; collector” (3 paratypes).

##### Diagnosis.

This species can be distinguished from all other described laraines by the following combination of characters: small size (ca. 4.5 mm), the presence of a shallow, wide, V-shaped groove across apical third of the pronotum (Fig. 53), and pronotum 1.3 mm long, and 1.5 mm wide, with posterolateral angles obtuse.

##### Intraspecific variation.

This species exhibits only minor variations in length, which ranges from 4.2 to 4.5 mm, and varies from a medium brown to light brown in color. Additionally, the parameres can be straight to slightly curved.

##### Distribution and habitat.

This species is known only from the type locality at Cerro de Neblina, Amazonas State, Venezuela (Fig. 2), see genus description for detailed habitat information.

##### Associated species.

The Cerro de Neblina endemic species *Neblinagena prima* was the only laraine elmid species collected in association with *Hypsilara royi*.

#### Neblinagena

Spangler, 1985

http://species-id.net/wiki/Neblinagena

Figs 5 , 54, 55, 56, 57 , 58, 59, 60, 61

##### Diagnosis.

**Figures 54–57.**
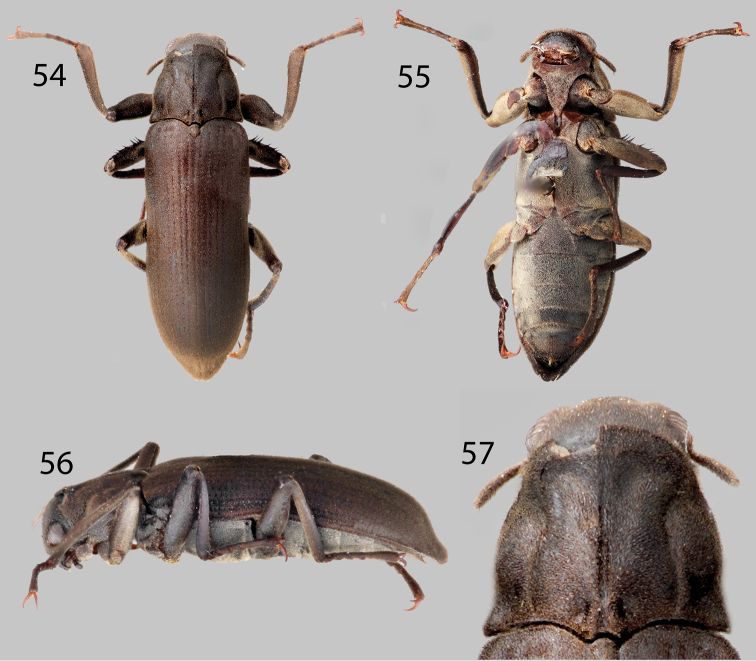
*Neblinagena doylei*: **54** Dorsal habitus **55** Ventral habitus **56** Lateral habitus **57** Pronotum, dorsal view.

*Neblinagena* can be distinguished from all other laraine genera by its large size (5.5–6.6 mm), slender body form (Fig. 54), dark color, and distinctive pronotum with a lateral longitudinal carina or arcuate-sinuate groove on basal third and two short, converging, prescutellar carinae, each with a deep pit laterally (Figs 57 and 61).

##### Distribution.

*Neblinagena* species are endemic to the Guiana Shield region in eastern Venezuela, though presumably they also occur across the borders in Guyana and Brazil (Fig. 5).

##### Habitat.

Based on the collecting events of specimens examined, *Neblinagena* spp. seem to prefer small, high elevation streams (>400m elevation), with rocks and forested cover. They have been found in leaf packs and clinging to rocks in the water flow. Their habits are most likely extremely similar to those of *Disersus* spp. and *Hexanchorus* spp.

**Figures 58–61.**
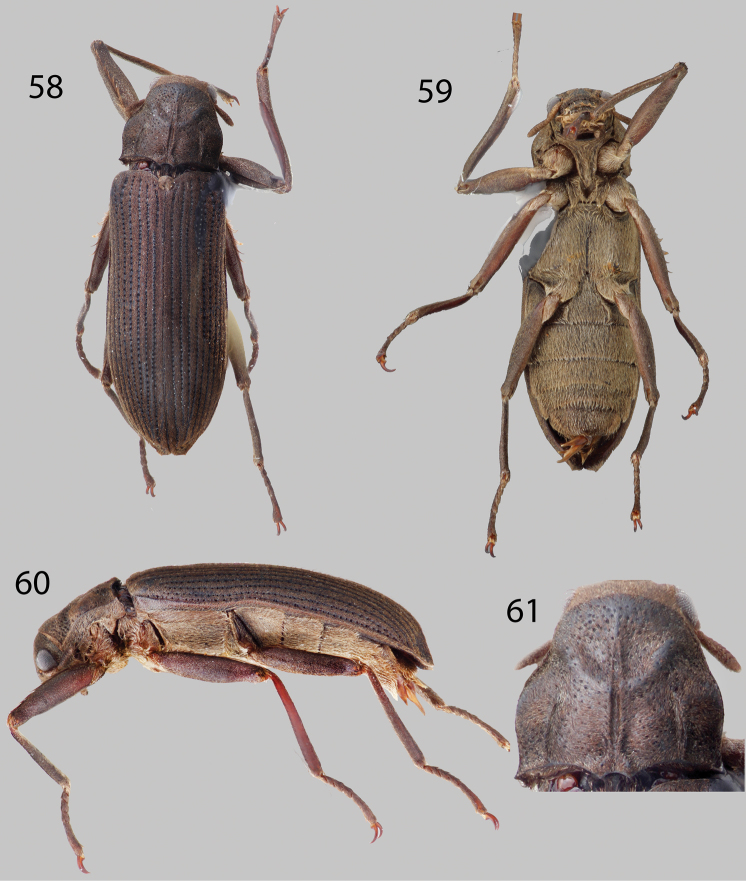
*Neblinagena prima*: **58** Dorsal habitus **59** Ventral habitus **60** Lateral habitus **61** Pronotum, dorsal view.

#### Nebilinagena doylei

Kodada & Jäch, 1999

http://species-id.net/wiki/Nebilinagena_doylei

Figs 5 , 54, 55, 56, 57

For complete species descriptions and genitalia illustrations, see Kodada and Jäch 1999.

##### Material examined.

**VENEZUELA: Bolívar State:** “SE-Venezuela, Bolívar State; Quebrada Wiwiparu, the stream; near Paratepui village, which; crosses the tourist pathway to Mt.” “Roraima, ca. 1000m a.s.l.; 31 Jan 1999; T. Derka & R. Reilmaier, lgt.” (1 Holotype NMW). “VENEZUELA: Bolívar State; 6°5'14.1"N, 61°23'55.8"W 400m; Piedra de la Virgen, 31.vii.2008; leg. A. Short, M. Garcia, L. Joly; AS-08-056, small forest stream” (4 specimens SEMC). “VENEZUELA: Bolívar State; 6°1'38"N, 61°23'41.1"W, 690m; Along La Escalera; 31.vii.2008; leg. A. Short, M. Garcia, L. Joly; AS-08-059; rocky stream” (4 specimens SEMC). “VENEZUELA: Bolívar State; 06°04'54.7"N, 61°23'52.7"W, 509 m; Along La Escalera, Highway 10; 14.vii.2010; leg. Short, Tellez, & Arias; VZ10-0714-01C” (2 specimens SEMC – frozen tissue collection) “VENEZUELA: Bolívar; Municipio Gran Sabana; El Pauji. 25/IV/2004 al 02/V/2004 J. Camacho; J. Perozo, Col.” “04°28'66"N, 61°35'38"W; 880msnm.” (9 specimens MALUZ). “Loc. VEN-16-2010, canyon; of the Rio Yuruen above; Uruyen village, Venezuela; Bolivar Province” “NP Canaima, 5°42'26.0"N; 62°28'13.7"W, alt. 658m; a.s.l. 16.i.2010; leg. T. Derka & M. Svitok (11 specimens CKB).

##### Diagnosis.

*Neblinagena doylei* can be distinguished from *Neblinagena prima* by its range, which does not overlap with the range of the latter. Additionally, in *Neblinagena doylei* the pronotal longitudinal carinae are entire and the oblique furrow of the pronotum forms a 30° angle relative to the median longitudinal furrow (Fig. 57), whereas in *Neblinagena prima* the pronotal lateral carinae are obscured anteriorly and the oblique furrows form a 45° angle relative to the median longitudinal furrow (Fig. 61). *Neblinagena doylei* is also slightly larger than its congener.

##### Intraspecific variation.

This species varies slightly in size (5.5–6.6 mm) and in coloration – from dark brown to black.

##### Distribution and habitat.

*Neblinagena doylei* can be found throughout the eastern part of Venezuela, in and around the Gran Sabana, in Bolívar and Amazonas States (Fig. 5). Specimens have been collected on flat, waterslide-like rocks in the La Escalera region.

##### Associated species.

This species is associated the Mount Roraima endemic species *Roraima carinata*. Other aquatic beetles collected at the same localities include: *Notionotus* spp., *Oocyclus* spp. (Hydrophilidae), and *Macrelmis* spp. (Elmidae: Elminae).

#### Neblinagena prima

Spangler, 1985

http://species-id.net/wiki/Neblinagena_prima

Figs 5 , 58, 59, 60, 61

For complete species description and genitalia illustrations see Spangler 1985.

##### Material examined.

**VENEZUELA: Amazonas State:**“VENEZUELA, T.F.Amaz. [Amazonas State]; Cerro de la Neblina; Camp X, 1690m; 0°54'N, 60°2'W; 12 February 1985” “Small sunlit stream; on rock surface in; falls between boulders; W.E. Steiner; collector” (1 paratype USNM). “VENEZUELA, T.F.Amaz. [Amazonas State]; Cerro de la Neblina; Camp XI, 1450m; 0°52'N, 65°58'W; 25–28 February 1985” “From leaf packs in; small rapid stream; P.J. & P.M. Spangler; & R.A. Faitoute; Seine of rapids in; small mountain stream” (1 holotype USNM, 1 paratype USNM). “Venezuela T.F. [Amazonas State]; Amazonas 600m; 18–24-X-1987” “Talud Cerro; Aracamuni; 1°29'N, 65°38'W” “Ex. Terramar” (1 specimen MIZA).

##### Diagnosis.

*Neblinagena prima* can be distinguished from *Neblinagena doylei* by its range, which does not overlap with the range of the latter. Additionally, the pronotal lateral carinae of *Neblinagena prima* are obscured anteriorly and the oblique furrows form a 45° angle relative to the median longitudinal furrow (Fig. 61), whereas in *Neblinagena doylei* the pronotal longitudinal carinae are entire and the oblique furrow of the pronotum forms a 30° angle relative to the median longitudinal furrow (Fig. 57).

##### Intraspecific variation.

This species varies slightly in size (6.0–6.3mm) and in coloration – from dark brown to black.

##### Habitat and distribution.

This species appears to be endemic to Cerro de Neblina, and is restricted to the highlands, unlike its congener, *Neblinagena doylei*, which is widespread in lower areas (<1000m) of Bolivar State, Venezuela (Fig. 5). *Neblinagena prima* was collected in leaf packs and between boulders in small mountain streams on Cerro de Neblina (Spangler 1985).

##### Associated species.

This species is associated with the other, highly unusual, Cerro de Neblina endemic, *Hypsilara royi*, and was collected in the same habitats.

#### Phanoceroides

Hinton, 1939

http://species-id.net/wiki/Phanoceroides

**Figures 62–65.**
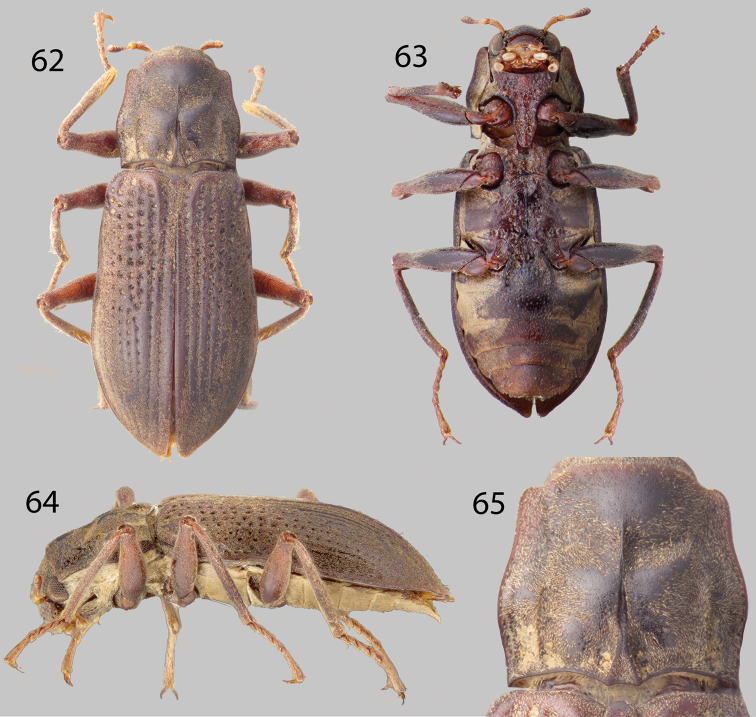
*Phanoceroides* sp. 1: **62** Dorsal habitus **63** Ventral habitus **64** Lateral habitus **65** Pronotum, dorsal view.

Figs 3 , 62, 63, 64, 65

##### Diagnosis.

*Phanoceroides* can be distinguished from all other genera of Larainae by the presence of a dense, silvery mat of setae on the ventral surface, or plastron (Fig. 63). They can be distinguished from members of the other elmid subfamily, Elminae, by the clubbed antennae, dense hairlike setae on the dorsum, and transverse procoxae (Fig. 63).

##### Distribution.

This genus is known from Manaus, Amazonas State, in the Amazon river basin in Brazil and from Tobogan de la Selva, Amazonas State, in the Orinoco River drainage in Venezuela, and probably occur throughout the Southern Venezuela and Northern Brazil. There have also been literature reports of *Phanoceroides* spp. from as far west as Cordillera de Vilcabamba, Peru, but this record has not been confirmed by me (Acosta et al. 1998).

##### Habitat.

*Phanoceroides* species have unique habitat requirements in the subfamily Larainae, in that they remain fully submerged and are found in the benthos of streams, as opposed to water-splashed surfaces in streams as others in Larainae (Hinton 1939).

##### Notes.

The genus *Phanoceroides* is rather interesting among the Larainae, in that while it bears superficial resemblance to beetles in the subfamily Elminae, in their fully aquatic habits and in the presence of a dense, hairy plastron on the ventral surface, anatomically, they are most similar to the Larainae, and therefore they are included in this work. Whether this is a case of convergent evolution or the retention of plesiomophic characters is unknown and it deserves further study, and this species may provide insights into the evolution of laraine Elmidae.

#### Phanoceroides sp. 1

Figs 3 , 62, 63, 64, 65

##### Material examined.

**VENEZUELA: Amazonas State:**“VENEZUELA, T.F. Amazonas; Puerto Ayacucho (40km S); El Tobogan, Caño Coromoto; 19 January 1989; leaf packs, upper riffles” “Collected by; P.J. Spangler; R.A. Faitoute & C.B. Barr” (22 specimens USNM).

##### Distribution and habitat.

In Venezuela, this species has only been collected at a single locality, Caño Coromoto, at El Tobogan de la Selva (Fig. 3). They were found in leaf packs and in benthic stream samples between the cascades. Additionally, specimens are known to come to UV lights.

##### Associated species.

No other species in Larainae has been collected with this *Phanoceroides* species, however, other aquatic beetles collected in the same habitat include: *Tyletelmis* spp., *Heterelmis* spp., *Gyrelmis* spp., *Neoelmis* spp., *Neolimnius* spp., *Pilielmis* spp. (Elmidae: Elminae), *Berosus* spp., *Chaetarthria* spp., *Phaenonotum* spp., *Derallus* spp., *Oocyclus* spp. (Hydrophilidae), *Laccodytes* spp. (Dytiscidae), Torridincollidae, *Lutrochus* spp. (Lutrochidae), Hydroscaphidae, and Meruidae.

##### Notes.

This species will be described in a later publication.

#### Phanocerus

Sharp, 1882

http://species-id.net/wiki/Phanocerus

Figs 6, 7, 8 , 66, 67, 68 , 69, 70, 71 , 72, 73, 74, 75, 76

##### Diagnosis.

This genus can be distinguished from all other genera of Larainae in Venezuela by its small size and pronotum which lacks a transverse groove (Fig. 68).

##### Distribution and habitat.

*Phanocerus* spp. can be found throughout Central America and northern South America. They are fast fliers and congregate in leaf packs and on water-splashed detritus in streams, waterfalls, and rivers.

**Figures 66–68.**
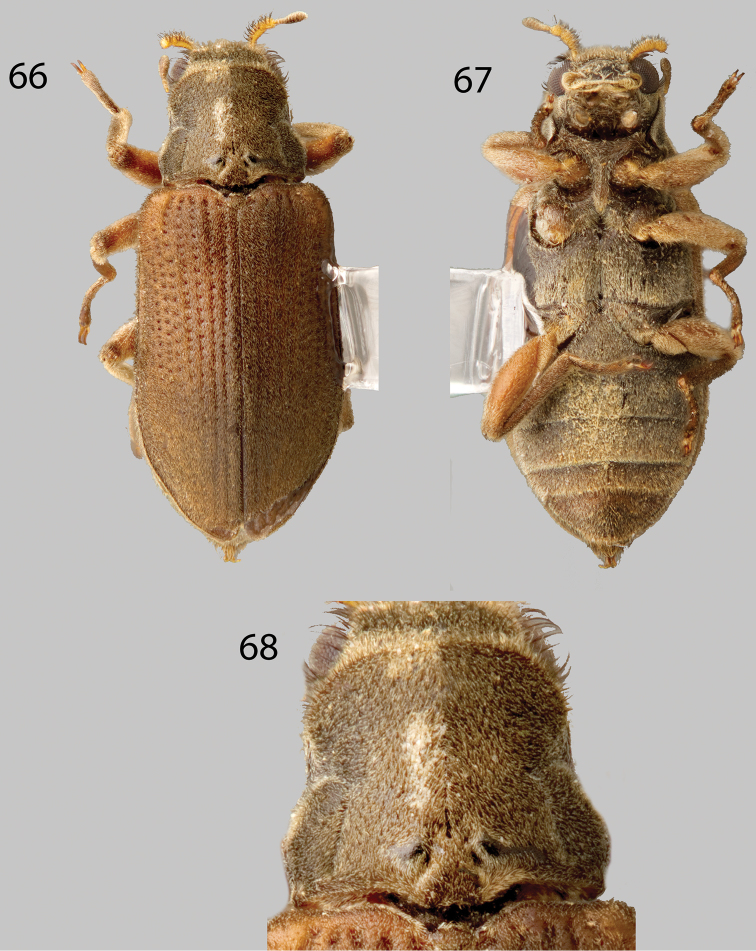
*Phanocerus clavicornis*: **66** Dorsal habitus **67** Ventral habitus **68** Pronotum, dorsal view

**Figures 69–71.**
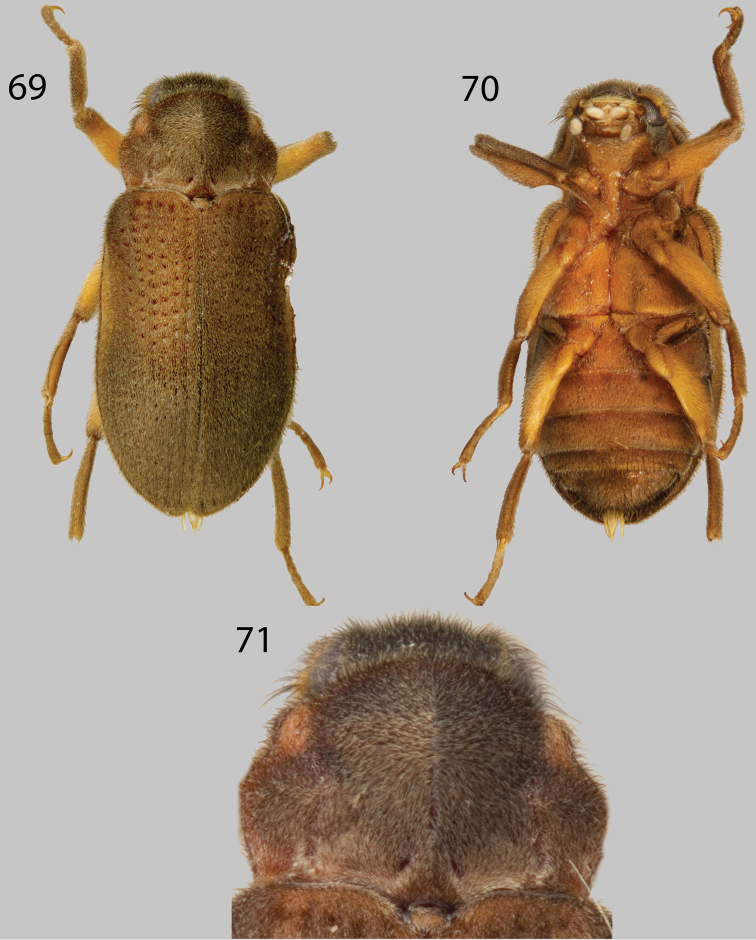
*Phanocerus congener*: **69** Dorsal habitus **70** Ventral habitus **71** Pronotum, dorsal view.

**Figures 72–76.**
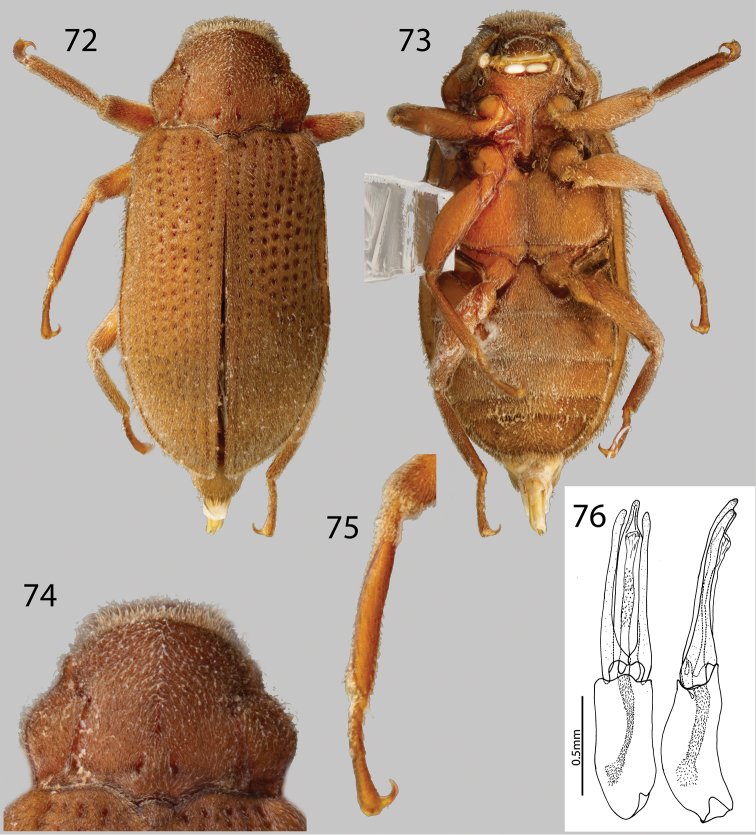
*Phanocerus rufus* sp. n.: **72** Dorsal habitus **73** Ventral habitus **74** Pronotum, dorsal view **75** Mesotibia, male **76** Aedeagus, dorsal and lateral views.

#### Phanocerus clavicornis

Sharp, 1882

http://species-id.net/wiki/Phanocerus_clavicornis

Figs 6 , 66, 67, 68

For complete redescription and genitalia illustrations, see Spangler and Santiago-Fragoso 1992.

##### Material examined.

**VENEZUELA: Amazonas State:** “Venezuela Exp[edition].; Territ. Amazonas; Upper Cunucunuma; Julian Apr. 28, 1950” “J. Maldonado; Capriles Coll.” (2 specimens USNM). “Isla de las; Hormigas TFA; 22-VIII-51” “ExpFcoVen; Alto Orinoco” (2 specimens USNM). **Barinas State:** “VENEZUELA: Barinas State; 8°49.334'N, 70°11.993'W, 203 m; nr. Santa Barbara; 15.vii.2009; leg Short, Gustafson, Camacho; Garcia & Inciarte; along river; margins/snags; VZ09-0715-04A” (5 specimens SEMC). “VENEZUELA: Barinas State; 8°49.334'N, 70°11.993'W, 203 m; nr. Santa Barbara; 15.vii.2009; leg. W. Shepard; gross sample; VZ09-0715-04Z” (6 specimens SEMC). **Bolívar State:** “Venezuela; Bolívar; Rio Caura; Salto Para; Playon; 23-XI-1978” “B. Bechyne; lgt.” (15 specimens MIZA). “Venezuela –BO[Bolívar State]; Kanarakuni; Alto Caura, 450 m” “10–13.9.1964; F.F. Yepez &; J. Bechyne lgt.” (7 specimens MIZA). “Venezuela- Boli-; var [Bolívar State]. Guri rio; Caroni. 100m; 16-XI-1966” “J. & B. Bechyne; E. Osuna” (2 specimens MIZA). “Venezuela- Boli-; var [Bolívar State]. Guri rio; Caroni. 100m.; 11-IV-1968” “J. Salcedo; col.” (1 specimen MIZA). “Venezuela- Boli-; var [Bolívar State]. Guri rio; Caroni. 100m.; 10-IV-1968” “J. Salcedo; col.” (1 specimen MIZA). **Mérida State:** “VENEZUELA: Mérida State; 8°57.205'N, 71°17.620'W, 88 m; W. of Tucani; 21.vii.2009; Gross sample from river; leg. Shepard; VZ09-0721-04Z” (26 specimens SEMC). “VENEZUELA: Mérida State; 8°49.749'N, 71°25.579'W, 68m; ca. 4 km E. Santa Elena; 28.i.2012; leg. Short, Arias, & Gustafson; River Habitats; VZ12-0128-04A” (1 specimen SEMC). **Monagas State:** “VENEZUELA: Monagas State; 10°02.289'N, 63°46.623'W, 686m; 1.ii.2010, Rio Caripicual, btw.; San Antonio & Mundo Nuevo; leg. Short, Garcia, & Joly; in flow; /kicknetting; VZ10-0201-04B” (1 specimen SEMC). “VENEZUELA: Monagas State; 10°04.306'N, 63°33.561'W, 464 m; Rio Aragua, nr. Rio Chiquito; 1.ii.2010; leg. A. Short; in flow, kick netting; VZ10-0201-01B” (1 specimen SEMC). “VENEZUELA: Sucre State [locality in Monagas State]; 10°00.840'N, 63°08.548'W, 38 m; Rio Azagua; 31.i.2010; leg. L. Joly; general river; collecting; VZ10-0131-03C” (1 specimen SEMC). **Táchira State:** “VENEZUELA: Táchira State; 7°35.038'N, 72°10.340'W, 472 m; El Tama National Park; 16.vii.2009; leg. Short, Sites, Garcia, Inciarte, Gustafson & Camacho; HG-vapor light; VZ09-0716-07A” (2 specimens SEMC). **Zulia State:** “VENEZUELA: Zulia State; 10.86041°N, 72.32210°W, 95m; Quebrada Reincito; 30.xii.2008; leg. A. Short & M. Garcia; VZ08-1230-01A; in riffle area” (2 specimens SEMC).

##### Diagnosis.

*Phanocerus clavicornis* can be separated from all other species of *Phanocerus* by the shape of the pronotum (Fig. 68). The pronotum is narrow in *Phanocerus clavicornis*, with narrow anterolateral angles, which are not explanate. Additionally, the setose patch at the base of the mesotibia is short (Fig. 66).

##### Intraspecific variation.

This species varies a great deal in color and size, from 2.0–2.7 mm in size, and from dark brown to medium brown in color. Occasional specimens can have a reddish cast, but this is rare.

##### Distribution and habitat.

The range of *Phanocerus clavicornis* is very large, and it is the most widely distributed laraine species in the Western Hemisphere. It occurs from Texas, in Southwestern United States, south to Amazonas State, Venezuela; it is also the most widely distributed in Venezuela, occurring throughout the country, excepting Los Llanos region in the central part of Venezuela (Fig. 6).That makes it the only species of laraine that crosses the Llanos and occurs on both the Guiana Shield and in the Northern Andes.

The habitat of *Phanocerus clavicornis* is similar to that of any other species of *Phanocerus*, it can be found in small to medium sized streams and rivers, in the leaf packs and on partially submerged detritus and woody debris.

##### Associated species.

Numerous species of aquatic Coleoptera have been collected with this common laraine elmid, however, they will not be listed here, since they are so widespread.

#### Phanocerus congener

Grouvelle, 1898

http://species-id.net/wiki/Phanocerus_congener

Figs 7 , 69, 70, 71

For complete redescription and genitalia illustrations, see Spangler and Santiago-Fragoso 1992.

##### Material examined.

**VENEZUELA: Aragua State:** “Venezuela: Aragua; near coast; 1 II 06 226 m; Rio Grande del Mérida; 10°27'N, 67°36'W” “A.E.Z. Short & P.J. Torres legs.” “Phanocerus congener; W.D. Shepard” (6 specimens SEMC). “VENEZUELA: Aragua State; 10.37319°N, 67.74250°W, 295m; Henri Pittier N.P.: Rio La Trilla; 4.i.2009; leg. A. Short; VZ09-0104-01B; kick netting” (12 specimens SEMC). “VENEZUELA: Aragua State; 10.39376°N, 67.79597°W, 130m; Henri Pittier N.P.: Rio Cumboto; 4.i.2009; leg. Short; leaf packs; & kick netting; VZ09-0104-02A” (35 specimens SEMC). “VENEZUELA: Aragua State; 10.48189°N, 67.60880'W, 152m; Rio Choroni; ca. 4km S. coast; 6.i.2009; leg. A. Short; VZ09-0106-03A: leaf packs” (34 specimens SEMC). “VENEZUELA: Aragua State: 10.37068°N, 67.59683°W, 1039m; H. Pittier N.P.: road to Choroni; 6.i.2009; Short, Miller, Camacho; Garcia; VZ09-0106-02X; stream” (1 specimen SEMC). “VENEZUELA: Aragua State; 10.48189°N, 67.60880°W, 152m; Rio Choroni; ca. 4 km S. coast; 6.i.2009; Short, Miller, Camacho; Garcia; VZ09-0106-03X” (1 specimen SEMC). “VENEZUELA: Aragua State; 10.39376°N, 67.79597°W, 130m; Henri Pittier N.P.: Rio Cumboto; 4.i.2009; leg. K.B. Miller; VZ09-0104-02A; river backwaters” (1 specimen SEMC). “VENEZUELA: Aragua; Par. Nac. Henri Pittier; Rancho Grande –Ocamure; 790 m, 20JUN1987, M.A. Ivie; leaf packs in waterfall” (4 specimens MAIC). **Barinas State:** “VENEZUELA: Barinas State; 8°49.334'N, 70°11.993'W, 203m; nr. Santa Barbara; 15.vii.2009; leg. W. Shepard; gross sample; VZ09-0715-04Z” (2 specimens SEMC). “VENEZUELA: Barinas State; 8°18.033'N, 70°45.201'W, 216m; River nr. Bum Bum; 15.vii.2009; leg. Short et al.; river margins; VZ09-0715-02A” (2 specimens SEMC). **Falcón State:** “VENEZUELA: Falcón State; 11°10.667'N, 69°33.695'W, 593 m; Cataratas del Hueque; 9.vii.2009; leg. Shepard; mud puddles/pools; gross sample; VZ09-0709-01Z” (1 specimen SEMC). “VENEZUELA: Falcón State; 11°10.667'N, 69°33.695'W, 593 m; Cataratas del Hueque; 9.vii.2009; Short & Gustafson; leafpacks/roots; at river margin; VZ09-0709-01Z” (4 specimens SEMC). **Trujillo State:** “VENEZUELA: Trujillo State; 9°11.840'N, 70°47.545'W, 1131m; ca. 2.5 km E. Monte Carmelo; 22.vii.2009; leg. W. Shepard; rocky stream after flooding; VZ09-0722-04Z; gross sample” (2 specimens SEMC). **Zulia State:** “VENEZUELA: Zulia; P.N. Perija: Tukuko; Rio Manantial; 16.VII.2008; A.E.Z. Short, leg.” “Phanocerus congener W.D. Shepard” (7 specimens SEMC). “VENEZUELA: Zulia State; 9°50.490'N, 72°49.310'W; Perija N.P. Tukuko: Rio Mantantial; 29.i.2009; Short, Garcia, Camacho; VZ09-0129-01A: gravel margin” (5 specimens SEMC). “VENEZUELA: Zulia, El; Tucuco (51 km S.O. de; Machiques). Trampa Ma-; laise. 01/03-II-1982” “Colectores; E. Inciarte; E. Rubio” (1 specimen MALUZ). “VENEZUELA: Zulia, El; Tucuco (51 km S.O. de; Machiques). 13.14-VIII-1982” “E. Rubio; colector” (1 specimen MALUZ). “Venezuela Zulia; Dtto. Maracaibo; San Jose de los; Altos 35 km N.; O. de Laberinto; 1400m. 16-vi-1989” “Colector; J. Camacho” (12 Specimens MALUZ). “VENEZUELA, Zulia, Mcpio. Rosario de; Perija, Rio Seco, 525; msnm. 16–18/II/1996” “Colectores; M. Garcia; D. Ascanio” (1 specimen MALUZ). “VENEZUELA: Zulia State; 9°50.513'N, 72°48.334'W, 252m; Perija N.P. Tukuko: Rio Tukuko; 29.i.2009; leg. Short, Garcia &; Camacho; VZ09-0129-02X” (1 specimen SEMC).

##### Diagnosis.

*Phanocerus congener* can be separated from all other species of *Phanocerus* by the following combination of characters: pronotum wide, 1.4x as wide as long, with wide anterolateral angles which are explanate (Fig. 71). Additionally, the setose patch at the base of the mesotibia of the male is long (Fig. 69).

##### Intraspecific variation.

Slight variations in size (2.1–2.5 mm) and color (light to dark brown) are common in *Phanocerus congener*.

##### Distribution and habitat.

This species has previously only been known from the Lesser Antilles, but is actually widespread in Venezuela, found throughout Western Venezuela in the Mérida Andes and the coastal ranges (Fig. 7). The habitat of *Phanocerus congener* is similar to that of other *Phanocerus* species, in partially submerged leaf packs and woody debris.

##### Associated species.

*Hexanchorus falconensis* sp. n. was collected at Cataratas del Hueque along with *Phanocerus congener*, in addition to *Heterelmis* spp., and *Microcylloepus* spp., *Lutrochus acuminatus* (Lutrochidae) (Maier and Short 2013), and larvae of Psephenidae.

#### Phanocerus rufus sp. n.

http://zoobank.org/94558BCC-E8D0-433C-927A-86D6545FABE6

http://species-id.net/wiki/Phanocerus_rufus

Figs 8 , 72, 73, 74, 75, 76

##### Type material.

**Holotype Male.**“VENEZUELA: Aragua State; 10.35669°N, 67.60645°W; Henri Pittier N.P.: Rio Castaño; Regesiva del Diablo; 6.i.2009; Short, Miller, Camacho, Garcia; VZ09-0106-01X” Holotype deposited in MIZA. **Paratypes (20):** Same data as holotype (9 Specimens SEMC). “VENEZUELA: Aragua State; 10.35669°N, 67.60645°W; Henri Pittier N.P.: Rio Castaño; Regesiva del Diablo; 6.i.2009; leg. A.E.Z. Short; VZ09-0106-01C; stream leaf packs” (9 specimens SEMC). “VENEZUELA: Aragua State; 10.35669°N, 67.60645°W; Henri Pittier N.P.: Rio Castaño; Regesiva del Diablo; 6.i.2009; A.E.Z. Short; VZ09-0106-01A; log in stream” (2 specimens SEMC). Paratypes will be deposited in: 2 in MIZA, 2 in MALUZ, 2 in USNM, 14 in SEMC.

##### Diagnosis.

*Phanocerus rufus* can be distinguished from all other species of *Phanocerus* by the combination of its large size (2.7–3.5 mm), reddish coloration (Fig. 72), distinctive pronotal shape, that is 1.6x as wide as long (Fig. 75), and the presence of a short setose patch at base of mesotibia (Fig. 74).

##### Description.

Holotype male. Body elongate, sub-parallel, moderately convex (Fig. 72). Total length 2.7 mm, greatest width 1.2 mm. Cuticle light reddish-brown, maxillary palpus, basal antennomeres, and legs testaceous (Fig. 73). Dorsal surface with dense, erect golden setae and denser golden, recumbent setae. Surface microreticulate, with dense, fine punctures.

Head moderately coarsely, densely punctate; punctures separated by their diameter; cuticle microreticulate. Clypeus with anterior margin rounded. Fronto-clypeal suture deep and curved. Labrum with anterior margin entire and gently rounded, narrower than clypeus; angle on each side obtuse, covered with setae approximately twice as long as setae on head. Eyes protruding only slightly laterally; separated by a distance about 3x the eye-width; bordered by short dark brown curved setae (“eyelashes”) that arise near dorsal and ventral sides of eyes and extend toward middle of eye, setae not as prominent as in other genera. Antenna 11 segmented, densely pubescent, slightly clubbed; basal two antennomeres with dense, medium-brown, brushy setae, thicker in width than proceeding antennomeres, with dense recumbent setae and dense brushy light brown setae. Antennal club of six antennomeres, compact, wide, quite thickened towards apex. Antennae very short, just barely reaching transverse groove of pronotum. Apical five antennomeres reddish-brown, with dense recumbent setae. Apical antennomere rounded.

Pronotum overall smooth, 1.6x as wide as long; widest at basal third; anterior width roughly two thirds the posterior width; anterior margin strongly convex over base of head; base tri-sinuate (Fig. 74). Pronotum with a sublateral groove, which joins deep anterolateral fovea near the anterior margin; anterolateral margins explanate; base with two small foveae anterior to scutellum; lateral margins strongly sinuate, nearly at right angles at basal third, strongly gibbous (Fig. 74); surface similarly punctate to head. Hypomeron oblique. Scutellum flat, broader than long; posterior angle square. Prosternum long in front of procoxae. Anterior margin reflexed ventrally. Prosternal process narrowly triangular, broad at base and tapering to apex; disc with strong median carina, lateral margins reflexed; apex strongly acuminate (Fig. 73). Mesoventrite short, depressed, with a deep, narrow, U-shaped depression for reception of apex of prosternal process (Fig. 73). Metaventrite with disc inflated on posterior three-fourths, finely punctate behind mesocoxae; with deep, impressed longitudinal groove on midline of disc, groove deepest and broadest on posterior third of disc (Fig. 73); with short, dense pubescence; cuticular surface of metaventrite finely microreticulate.

Elytra more than 3.4x as long as pronotum; lateral margins slightly sinuate; humeri and base adjacent to scutellum slightly gibbous; lateral margins smooth; apex smoothly rounded. Each elytron with 10 coarse striae formed by a row of large, coarse punctures separated by more than three times their diameter; striae slightly impressed becoming narrower and more shallow towards the apex; strial punctures coarse basally, becoming progressively finer towards apex; striae 3 and 4 not converging sub-apically; intervals flat (Fig. 72). Elytral surface with dense, golden recumbent pubescence and dense, erect hair-like setae.

Legs thin and short. Pro-, meso- and metatibiae lacking fringe of tomentum. Protibia glabrous ventrally, tomentose dorsally. Mesotibia with short basal setose patch and short apical setose patch (Fig. 75). Metatibia tomentose. Apical tarsomere of all legs entirely pubescent.

Abdomen with five ventrites, all ventrites pubescent, covered with fine, golden setae (Fig. 73). First ventrite lacking longitudinal carina behind metacoxae; cuticle densely covered with short, recumbent setae. Ventrite 4 lacking V-shaped carina. Last ventrite subtruncate, medially with patch of dense, long, dark brown setae. Aedeagus slightly curved, with parameres nearly as long as aedeagus (Fig. 76). Internal sac quite visible in slide mount, densely lined with spicules (Fig. 76).

**Female.** Externally similar to male except slightly larger in size, protibiae slightly less curved than those of male. Metaventral disc not as deeply and less concave. Apical abdominal ventrite with less dense setae than male.

##### Intraspecific variation.

This species varies slightly in color, from dark reddish-brown to medium reddish-brown, length (2.7–3.5 mm), and degree of setation.

##### Distribution and habitat.

This species has only been collected at the type locality at Rio Castaño, a small river in cloud forest on the interior slopes of Henri Pittier National Park, in Aragua State, Venezuela (Fig. 8). They were collected on logs in the stream and in leaf packs.

##### Etymology.

This species is named *Phanocerus rufus*, in reference to the slightly reddish cast of the cuticle.

##### Associated species.

*Phanocerus rufus* was found in crevices on submerged logs along with the lutrochid, *Lutrochus acuminatus* (Maier and Short 2013).

##### Other material examined

(Not Assigned to species – all female, likely new species).

**Population 1: VENEZUELA: Monagas State:**“VENEZUELA: Monagas State; 10°10.322'N, 63°33.315'W; 1110m; Gauchero Cave National Park; 20.vii.2010; leg. Short, Tellez, Arias; along stream; VZ10-0720-02A” (2 specimens SEMC).

**Population 2: Trujillo State:** “VENEZUELA: Trujillo State; 9°11.935'N, 70°45.233'W, 1601m; ca. 6 km E Monte Carmelo; 22.vii.2009; leg. W. Shepard; VZ09-0722-03Z” (4 specimens SEMC).

**Population 3: Aragua State:** “VENEZUELA: Aragua; 19 km. N. Maracay; 2 July 1986; R.S. Miller colr” (1 specimen MAIC).

**Population 4: Barinas State:** “VENEZUELA: Barinas; nr. Alta Mira; 5 July 1986; R.S. Miller colr.; Riparian woodland” (1 specimen MAIC).

#### Pharceonus

Spangler & Santiago-Fragoso, 1992

http://species-id.net/wiki/Pharceonus

Figs 5, 8 , 77, 78, 79, 80 , 81, 82, 83, 84 , 85, 86, 87

##### Diagnosis.

The genus *Pharceonus* can be distinguished from all other genera of Laraine Elmidae that occur in South America by its small size and distinctive pronotum. All *Pharceonus* species are small (3.1–4.3 mm), but generally larger than the similar genus *Phanocerus*, in various shades of medium to dark brown, with a pronotum that has a distinctive transverse bisinuate impression at the apical third and a transverse, bisinuate impression subbasally (Fig. 83). These two impressions form two gibbosities on the basal two thirds of the pronotum (Fig. 83).

**Distribution.** *Pharceonus* species occur throughout southern Central America and northern South America, from Costa Rica south to Peru (pers. obs.).

**Habitat.** Members of this genus can be found on roots and woody debris in small to medium forested streams and seeps, as well as in the benthos, among gravel and sometimes on on rocky substrate (Spangler and Santiago-Fragoso 1992).

**Figures 77–80.**
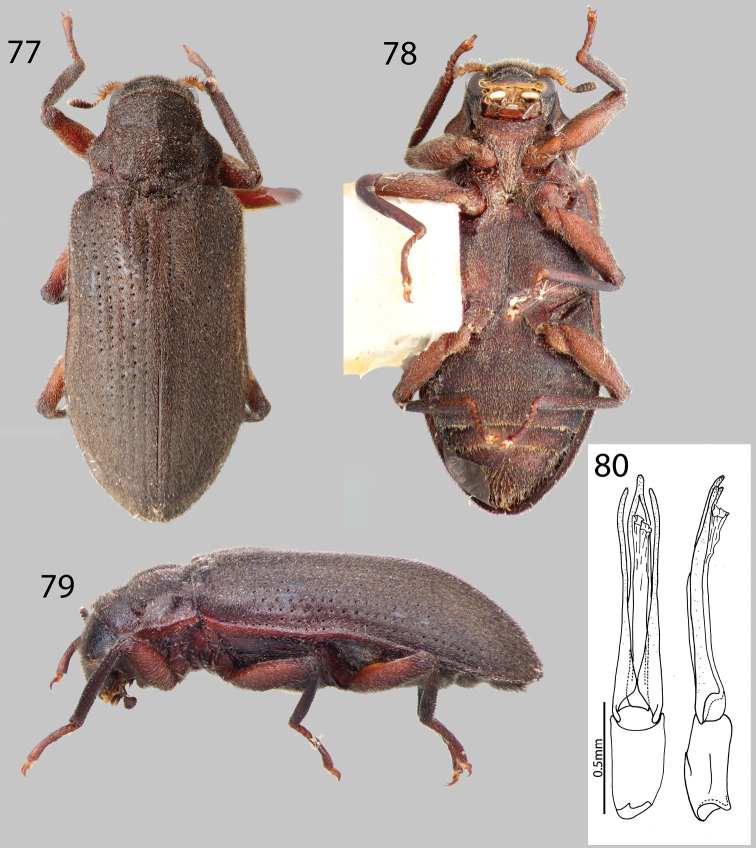
*Pharceonus ariasi* sp. n.: **77** Dorsal habitus **78** Ventral habitus **79** Lateral habitus **80** Aedeagus, dorsal and lateral views.

**Figures 81–84.**
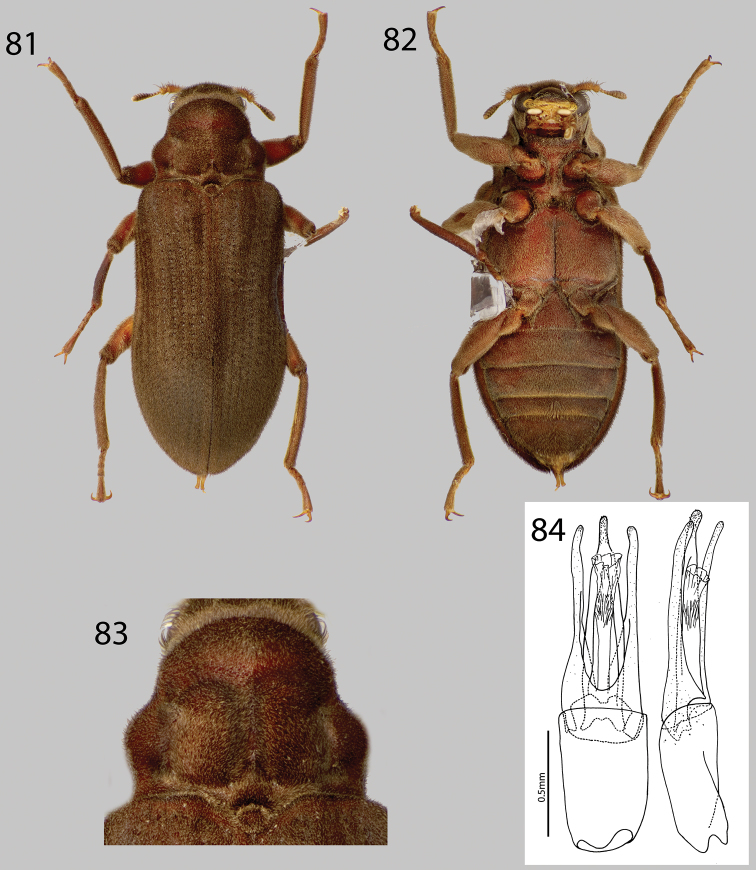
*Pharceonus grandis* sp. n.: **81** Dorsal habitus **82** Ventral habitus **83** Pronotum, dorsal view **84** Aedeagus, dorsal and lateral views.

**Figures 85–87.**
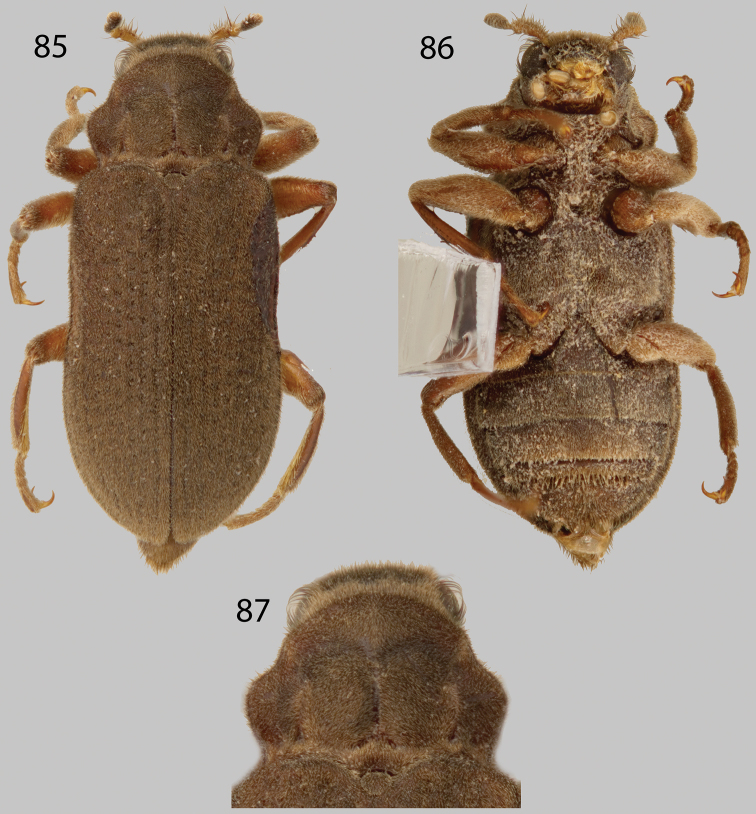
*Pharceonus volcanus*: **85** Dorsal habitus **86** Ventral habitus **87** Pronotum, dorsal view.

#### Pharceonus ariasi sp. n.

http://zoobank.org/C8211343-31E1-4EBC-AD20-E503849B458E

http://species-id.net/wiki/Pharceonus_ariasi

Figs 8 , 77, 78, 79, 80

##### Type material.

**Holotype Male.**“Venezuela: Mérida State; 8°52.423'N, 70°37.611'W, 1616m; Cascada Velo de la Novia; 24.i.2012; leg. Short, Arias, & Gustafson; logs and kicknetting; VZ12-0124-01B” Holotype deposited in MIZA. **Paratypes (18):**“VENEZUELA: Mérida State; 8°52.423'N, 71°37.611'W, 1616 m; Cascada Velo de la Novia; 19.vii.2009; leg. W. Shepard; gross sample; VZ09-0719-01Z” (4 specimens SEMC). “VENEZUELA: Mérida State; 8°51.933'N, 70°37.131'W, 1682m; ca. 12 km SE of Santo Domingo; leg. Short, Arias, & Gustafson; 22.i.2012; log and stick jams; in river; VZ12-0122-03B” (8 specimens SEMC). “VENEZUELA: Mérida State; 8°51.933'N, 71°37.131'W, 1682m; ca. 12 km SE Santo Domingo; 19.vii.2009; leg. W. Shepard; gross sample; VZ09-0719-02Z” (6 specimens SEMC). Paratypes will be deposited in: 2 in MIZA, 2 in MALUZ, 2 in USNM, 12 in SEMC.

##### Diagnosis.

This species can be distinguished from all other species of *Pharceonus* by its large size (3.5–4.1mm), dark brown color (Fig. 77), narrow genitalia (aedeagus + phallobase) (6x as long as wide) (Fig. 80), and pronotum with only a shallow bisinuate transverse impression across apical third (Fig. 77).

##### Description.

Holotype male. Body elongate, sub-parallel, moderately convex. Total length 3.3 mm, greatest width 1.3 mm. Cuticle dark brown, base of maxillary palpus, six basal antennomeres, femora, and tarsi testaceous (Fig. 78). Dorsal surface with dense, erect brownish hair-like setae and denser and finer, golden, recumbent setae (Fig. 77). Surface microreticulate, with dense fine punctures.

Head moderately coarsely, densely punctate; punctures separated by their diameter; cuticle microreticulate. Clypeus with anterior margin truncate. Fronto-clypeal suture deep and slightly curved. Labrum with anterior margin entire and straight; angle on each side rounded, covered with golden setae approximately twice as long as setae on head. Eyes protruding only slightly laterally; separated by a distance about three times the eye-width; bordered by long dark brown curved setae (“eyelashes”) that arise near dorsal and ventral sides of eyes and extend toward middle of eye, setae not as prominent as in other genera. Antenna 11 segmented, pubescent, slightly clubbed; basal two antennomeres with long, brown, hair-like setae, thicker in width than proceeding antennomeres, with dense recumbent setae and dense brushy light brown setae (Fig. 77). Antennal club of six antennomeres, compact, thickened towards apex. Antennae short, reaching transverse groove of pronotum. Apical five antennomeres dark brownish black, with dense recumbent setae. Apical antennomere rounded.

Pronotum heavily sculptured, wider than long (1.1 mm and 1.0 mm, respectively); widest at basal third; anterior width two thirds the posterior width; anterior margin strongly convex over base of head; base tri-sinuate (Fig. 77). Pronotum with a shallow sublateral depression on each side extending from a deep anterolateral fovea near the anterior margin towards the base, where it is joined to sub-basal, transverse and bisinuate shallow depression; a short medial longitudinal and superficial groove joins the sub-basal and transverse depression with a strong tranverse and bisinuate depression at apical third; surface similarly punctate to head (Fig. 77). Pronotal base with strong median gibbosity anterior to scutellum. Hypomeron oblique (Fig. 79). Scutellum flat, broader than long, elevated posteriorly; posterior angle obtuse. Prosternum long in front of procoxae; with tuft of long, golden setae apicomedially. Anterior margin reflexed ventrally. Prosternal process scutiform, broad at base and tapering to apex; disc slightly impressed, lateral margins reflexed; middle convex; apex acuminate (Fig. 78). Mesoventrite short, depressed, with a deep, narrow, V-shaped depression for reception of apex of prosternal process. Metaventrite with disc inflated on posterior three-fourths, finely punctate behind mesocoxae; with deep, impressed longitudinal groove on midline of disc, groove deepest and broadest on posterior third of disc; with short, dense pubescence; cuticular surface of metaventrite finely microreticulate (Fig. 78).

Elytra more than three times as long as pronotum; lateral margins slightly sinuate; humeri and base adjacent to scutellum strongly gibbous; lateral margins smooth; apex rounded (Fig. 79). Each elytron with 10 coarse striae formed by a row of deep punctures well separated by more than three times their diameter; striae slightly impressed becoming narrower and more shallow towards the apex; strial punctures coarse basally, becoming progressively finer towards apex; striae 3 and 4 converging sub-apically; intervals flat (Fig. 77). Elytral surface with dense, golden pubescence.

Legs thin and short. Pro-, meso- and metatibiae with very short fringe of tomentum extending from about mid-tibia to nearly the tip. Surface of legs entirely pubescent, including mesotibiae. Mesotibia with small glabrous patch basally. Apical tarsomere of all legs with glabrous patch dorsally.

Abdomen with five ventrites. First ventrite distinctly carinate adjacent to metacoxae; carinae extending longitudinally behind metacoxae for almost entire length of first ventrite; cuticle densely covered with short, recumbent setae (Fig. 78). Ventrite IV with median V-shaped carina; carina covered with thicker setae than rest of ventrite. Last ventrite subtruncate, covered with dense, long, dark brown setae. Genitalia (aedeagus + phallobase wide, 6x as long as wide, slightly curved (Fig. 80).

**Female.** Externally similar to male except slightly larger in size, elytral gibbosities more producted, and elytral sutural stria slightly inflated in apical third. Protibiae slightly less curved than those of male. Metaventral disc not as deeply and less concave. Abdominal ventrite IV lacking V-shaped carina. Apical abdominal ventrite with less dense setae than male.

##### Intraspecific variation.

This species varies slightly in color, from dark brown to medium brown, length (3.5–4.1 mm), and degree of setation.

##### Etymology.

The specific epithet “*ariasi*” is a patronym in honor of Mr. Quintin Arias, who helped to collect many of the specimens of this species.

##### Distribution and habitat.

This species is limited to the type locality at Cascada Velo de la Novia and from a nearby stream 12 km southeast of Santo Domingo, in Mérida State, Venezuela (Fig. 8). The specimens were found clinging to waterlogged woody debris in the river.

##### Associated species.

No other laraine species were collected with *Pharceonus ariasi*.

#### Pharceonus grandis sp. n.

http://zoobank.org/1394A6F1-8545-41DF-9CA5-AD5B8E8A6FAF

http://species-id.net/wiki/Pharceonus_grandis

Figs 5 , 81, 82, 83, 84

##### Type material.

**Holotype Male.**“Venezuela: Mérida State; 8°38.006'N, 71°09.782'W, 2037m; Monte Zerpa area; 20.vii.2009; leg. W. Shepard; stream margin; VZ09-0720-01Z” Holotype deposited in MIZA. **Paratypes (20): VENEZUELA: Mérida State:** Same data as Holotype (14 specimens SEMC). “Venezuela: Mérida State; 8°38.006'N, 71°09.782'W, 2037m; Monte Zerpa area; 20.vii.2009; leg. Short, Sites, Gustafson, &; Camacho; stream margin/pools; VZ09-0720-01A/L-1098” (1 specimen SEMC). “VENEZUELA: Mérida State; 8°35.355'N, 71°13.926' W 1646m; N. of Ejido, Rt. 4 river x-ing; 10.vii.2009; leg. Shepard; gross sample; VZ09-0720-02Z” (4 specimens SEMC). “VENEZUELA: Mérida State; 8°48.725'N, 70°47.057'W, 3012m; ca. 6 km E. Laguna Mucubaji; by Hotel Los Frailes; 19.vii.2009; W. Shepard; VZ09-0719-03Z; gross sample” (1 specimen SEMC). Paratypes will be deposited in: 2 in MIZA, 2 in MALUZ, 2 in USNM, 14 in SEMC.

##### Diagnosis.

This species can be distinguished from all other species of *Pharceonus* by its large size (3.5–4.3 mm), slightly reddish color (Fig. 81), wide genitalia (aedeagus + phallobase) (3.7x as long as wide) (Fig. 84), and pronotum with a deep and strongly bisinuate transverse impression across apical third (Fig. 83).

##### Description.

Holotype male. Body elongate, sub-parallel, moderately convex. Total length 3.7 mm, greatest width 1.6 mm. Cuticle dark reddish-brown, base of maxillary palpus, six basal antennomeres, femora, and tarsi lighter reddish-brown (Fig. 82). Dorsal surface with dense, erect brownish hair-like setae and denser and finer, golden, recumbent setae. Surface microreticulate, with dense fine punctures.

Head moderately coarsely, densely punctate; punctures separated by their diameter; cuticle microreticulate. Clypeus with anterior margin rounded. Fronto-clypeal suture deep and curved. Labrum with anterior margin entire and gently rounded; angle on each side obtuse, covered with setae approximately twice as long as setae on head. Eyes protruding only slightly laterally; separated by a distance about 3.5x the eye-width; bordered by long dark brown curved setae (“eyelashes”) that arise near dorsal and ventral sides of eyes and extend toward middle of eye, setae not as prominent as in other genera. Antenna eleven segmented, pubescent, slightly clubbed; basal two antennomeres with long, brown, hair-like setae, thicker in width than proceeding antennomeres, with dense recumbent setae and dense brushy light brown setae. Antennal club of six antennomeres, compact, thickened towards apex (Fig. 81). Antennae short, reaching transverse groove of pronotum (Fig. 81). Apical five antennomeres dark brownish black, with dense recumbent setae. Apical antennomere rounded.

Pronotum heavily sculptured, as wide as long (0.9 mm and 0.9 mm, respectively); widest at basal third; anterior width two thirds the posterior width; anterior margin strongly convex over base of head; base tri-sinuate (Fig. 83). Pronotum with a sublateral depression on each side extending from a deep anterolateral fovea near the anterior margin towards the base, where it is joined to sub-basal, transverse and bisinuate depression; a short medial longitudinal and superficial groove joins the sub-basal and transverse depression with a strong tranverse and bisinuate depression at apical third; surface similarly punctate to head. Pronotal base with strong median gibbosity anterior to scutellum (Fig. 83). Hypomeron oblique. Scutellum slightly convex, broader than long, elevated posteriorly; posterior angle square. Prosternum long in front of procoxae; with tuft of long, golden setae apicomedially. Anterior margin reflexed ventrally. Prosternal process narrowly triangular, broad at base and tapering to apex; disc slightly impressed, lateral margins reflexed; middle convex; apex acuminate (Fig. 82). Mesoventrite short, depressed, with a deep, narrow, V-shaped depression for reception of apex of prosternal process. Metaventrite with disc inflated on posterior three-fourths, finely punctate behind mesocoxae; with deep, impressed longitudinal groove on midline of disc, groove deepest and broadest on posterior third of disc; with short, dense pubescence; cuticular surface of metaventrite finely microreticulate (Fig. 82).

Elytra more than three times as long as pronotum; lateral margins slightly sinuate; humeri and base adjacent to scutellum strongly gibbous; lateral margins smooth; apex rounded. Each elytron with ten coarse striae formed by a row of large punctures well separated by more than three times their diameter; striae slightly impressed becoming narrower and more shallow towards the apex; strial punctures coarse basally, becoming progressively finer towards apex; striae 3 and 4 converging sub-apically; intervals flat (Fig. 81). Elytral surface with dense, golden pubescence.

Legs thin and short (Fig. 81). Pro-, meso- and metatibiae with very short fringe of tomentum extending from about mid-tibia to nearly the tip. Surface of legs entirely pubescent, including mesotibiae. Mesotibia with small glabrous patch basally. Apical tarsomere of all legs with glabrous patch dorsally.

Abdomen with five ventrites. First ventrite distinctly carinate adjacent to metacoxae; carinae extending longitudinally behind metacoxae for almost entire length of first ventrite; cuticle densely covered with short, recumbent setae (Fig. 82). Ventrite IV with median V-shaped carina; carina covered with thicker setae than rest of ventrite (Fig. 82). Last ventrite subtruncate, covered with dense, long, dark brown setae. Genitalia (aedeagus + phallobase wide, 3.7× as long as wide, gently curved (Fig. 84).

**Female.** Externally similar to male except slightly larger in size, elytral gibbosities more producted, and elytral sutural stria slightly inflated in apical third (Fig. 81). Protibiae slightly less curved than those of male. Metaventral disc not as deeply and less concave. Abdominal ventrite 4 lacking median V-shaped carina. Apical abdominal ventrite with less dense setae than male.

##### Intraspecific variation.

This species varies slightly in color, from dark reddish-brown to medium reddish-brown, length (3.5–4.2 mm), and degree of setation.

##### Distribution and habitat.

*Pharceonus grandis* has been collected only in the Mérida Andes in Venezuela (Fig. 5), at stream margins and in bulk benthic samples.

##### Etymology.

*Pharceonus grandis* is named after the Latin “grandis”, meaning large, referring to its large size.

##### Associated species.

No other laraine species were collected with *Pharceonus grandis*.The following aquatic beetle taxa were collected at the same localities as *Pharceonus grandis*: *Andogyrus* spp. (Gyrinidae), *Andonectes* spp. (Dytiscidae), *Enochrus* spp. (Hydrophilidae), *Hydraena* spp. (Hydraenidae).

#### Pharceonus volcanus

Spangler & Santiago-Fragoso, 1992

http://species-id.net/wiki/Pharceonus_volcanus

Figs 5 , 85, 86, 87

See Spangler and Santiago-Fragoso 1992 for complete species description and genitalia illustrations.

##### Material examined.

**PANAMA: Chiriqui:** “PANAMA, Chiriqui; Volcan (26km W); 1380 m, small brook; at culvert, 3 June1983” “Collectors; P.J. Spangler; R.A. Faitoute; W.E. Steiner” (1 Holotype USNM). **VENEZUELA: Zulia State:** “VENEZUELA: Zulia; SW of Machiques; 31 XII 05; 435m; 10°03'N, 72°43'W; trib[utary] of Rio Negro” “A.E.Z. Short; P.J. Torres; collectors” (5 specimens SEMC). “VENEZUELA: Zulia State; 10°03.058'N, 72°42.974'W; 435m; Perija National Park; Toromo; 31.xii.2005; leg. A.E.Z. Short; Small stream & seep; AS-06-001” (3 specimens SEMC).

##### Diagnosis.

This species can be distinguished from all other species of *Pharceonus* by its small size (3.1–3.5 mm), dark brown color, genitalia (aedeagus + phallobase) of medium width (4.3× as long as wide), and pronotum with only a shallow transverse impression across apical third (Fig. 87).

##### Intraspecific variation.

This species varies slightly in color, from dark brown to medium brown, and size, from 3.1–3.5 mm.

##### Distribution and habitat.

This species is found on roots in small to medium forested streams, and on roots in seeps in the Sierra de Perija in Venezuela (Fig. 5). This appears to be the southern extent of its range. Outside of Venezuela, this species is known from as far north as Costa Rica. Specimens have been collected in leaf packs and in the benthos in shallow streams.

##### Associated species.

This species was collected at the same sites as following taxa: *Oocyclus* spp., *Anacaena* spp., *Enochrus* spp., *Notionotus* spp., and *Chasmogenus* spp. (Hydrophilidae), and Hydraenidae.

#### Potamophilops

Grouvelle, 1896

http://species-id.net/wiki/Potamophilops

**Figures 88–92.**
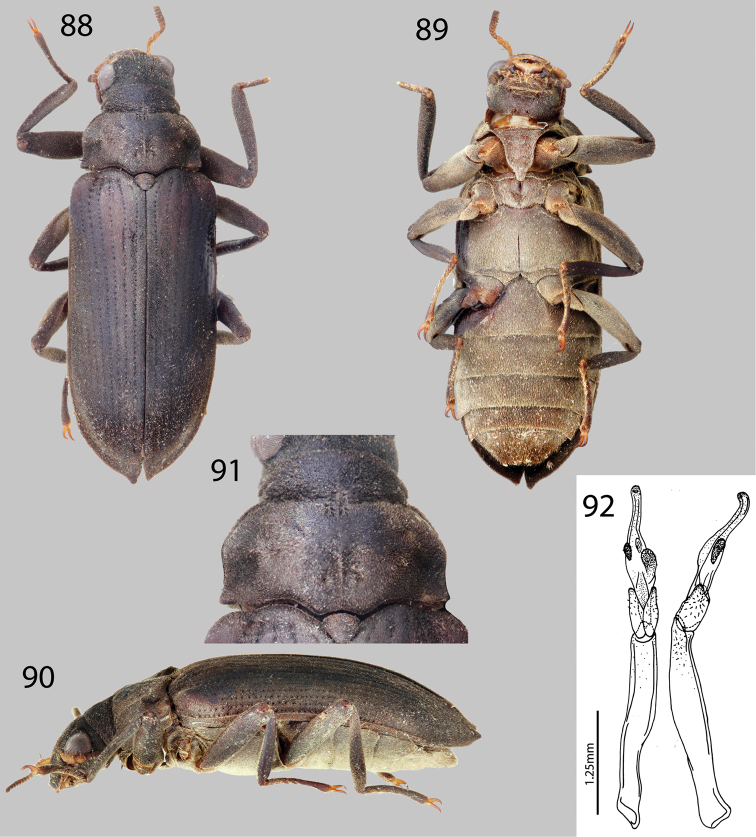
*Potamophilops bostrychophallus* sp. n.: **88** Dorsal habitus **89** Ventral habitus **90** Lateral habitus **91** Pronotum, dorsal view **92** Aedeagus, dorsal and lateral views.

Figs 1 , 88, 89, 90, 91, 92

##### Diagnosis.

The genus *Potamophilops* can be distinguished from all other New World laraine genera by its large size (>5.8 mm), the absence of an accessory elytral stria (Fig. 88), and the presence of a deep, transverse impression on the anterior third of the pronotum (Fig. 91).

##### Distribution.

*Potamophilops* has been recorded from as far south as northern Argentina, Mato Grosso and São Paulo States, Brazil (Spangler and Santiago-Fragoso 1987; Vanin and Costa 2011), and Paraguay (USNM, pers. obs.) (*Potamophilops cinereus*), and from as far north as Taquaruçú, Tocantins state, Brazil (*Potamophilops bragaorum* Fernandes & Hamada, 2012). Here I report the first records of *Potamophilops* from Venezuela (Fig. 1).

##### Habitat.

Fernandes and Hamada report *Potamophilops bragaorum* from a small mountainous stream in the Cerrado of Brazil. They were collected in cascades, on submerged logs, and on riparian vegetation, similar to *Disersus* spp. and *Pseudodisersus* spp. (Fernandes and Hamada 2012).

#### Potamophilops bostrychophallus sp. n.

http://zoobank.org/B8F60D69-9871-4C87-9790-495791444BFE

http://species-id.net/wiki/Potamophilops_bostrychophallus

Figs 1 , 88, 89, 90, 91, 92

##### Type material.

Holotype Male. “Venezuela, Bo-; livar, Kanara-; kuni 450m; 4-II-1967” “F. Fernandez Y; A.D. Asco”. Holotype deposited in MIZA. **Paratypes (37): VENEZUELA: Bolívar State:** “Venezuela, Bolívar; El Bochincho Res.; Forestal Imataca; 200m, 6–13-XII-74” “Expedicion Instituto; Zoologia Agricola; Fac. Agronomia; U.C.V.” (7 specimens MIZA). “El Playon; Rio Caura; Venezuela, Boli-; var [Bolívar State]. 140m; 23-XI-1978” “B. Bechyne leg.” (1 specimen MIZA). “El Playon; Rio Caura; Venezuela, Boli-; var [Bolívar State]. 100m; 8–10-XI-1978” “E. Osuna; J. Clavijo leg.” (1 specimen MIZA). “Venezuela, Bo-; livar, Kanara-; kuni 450m; 3-II-1967” “F. Fernandez Y; A.D. Asco” (1 specimen MIZA). “VENEZUELA Bolívar; cr. San Rafael de Pendare; 06°06'27"N, 67°05'34"W; 17-vi-2000 M. Gaiani; P.; Freytag; Q. Arias” (1 specimen MIZA). “Venezuela- Boli-; var.” “El Bochinche; Res. Forestal; Imataca 200m; 16–18-V-85” “Exp. Instituto; Zool. Agricola” (1 Specimen MIZA). “Venezuela – Bolívar; Rio Caura; Salto Para; Via Playon; 23-X-78” “B. Bechyne; leg.” (2 specimens MIZA). “VENEZUELA, Bolivar; Rio Caura, El Playon env.; 06°19'33.2"N, 064°31'373"W, 27.11.2011; Čiamporová-Zaťovičová & Čiampor Jr lgt.” (23 specimens FCC). Paratypes will be deposited in: 1 in MALUZ, 2 in USNM, 2 in SEMC, 9 in MIZA, 23 FCC.

##### Diagnosis.

This species can be distinguished from all other species of *Potamophilops* by the following combination of characters: its small size (<6.8 mm TL); the presence of distinct but subtle postmetacoxal carinae (Fig. 89); and the distinctive aedeagus, with a curled apex (Fig. 92).

##### Description.

Elongate, subparallel, moderately convex dorsally (Fig. 88). Length, 6.1 mm; width, 2.2 mm. Black dorsally; antennomeres I and II testaceous; antennomeres II–XI black. Ventral surface black except maxillary palpomeres I and II, labial palpi, labium, maxillae, coxae, trochanters, bases of femora, tarsal claws, and a small area behind each metacoxa on first abdominal ventrite light brown; mesotibiae medium reddish-brown (Fig. 89).

Head finely, densely punctate; punctures separated by distance equal to about half their diameter. Eyes large, hemispherical. Clypeus shallowly arcuately emarginate anteriorly. Labrum, especially on anterior half, densely punctate; anterior margin shallowly and broadly emarginate, and densely fringed with long, fine, golden, hair-like setae; anterolateral angles rounded and greatly expanded laterally.

Pronotum widest at base; length, 1.5 mm; width, 1.7 mm; sides arcuate; anterolateral angles obtuse, with distinct constriction posterolaterally of each angle resulting from deep transverse impression across apical third of pronotum (Fig. 91); apex arcuate; base strongly bisinuate; with a shallow fovea on each side of midline a short distance in front of scutellum; posterolateral angles obtuse; with a deep, broad depression adjacent to each angle, angled reflexed dorsally; surface with deep transverse impression across apical third; midline convex behind transverse impression (Fig. 91); discal area finely densely punctate, punctures separated by a distance equal to or less than their diameter.

Prosternum very short in front of procoxae. Prosternal process wide, elongate, apex with median process extending posteriorly further than sides (Fig. 89). Mesoventrite with moderately deep U-shaped depression for reception of apex of prosternal process. Metaventrite convex on each side of midline, depressed, with a glabrous line posteromedially between metacoxae (Fig. 89); surface microreticulate and punctate; punctures on convex surface fine and dense, separated by a distance equal to or less than their diameter; punctures sparser laterally.

Legs long and slender. Procoxae and metacoxae moderately widely separated; mesocoxae slightly more widely separated (Fig. 89). Mesotibiae entirely glabrous, except a very narrow strip on medial (lower) surface, with sparse coarse punctures (Fig. 90). Metatibia covered with dense pubescence. Tarsal claws long and stout.

Elytron with 10 rows of fine, nearly confluent punctures, punctures separated by a distance less than their diameter; intervals finely densely punctate, punctures separated by distance about equal to their diameter and obscured by dense pubescence; humeral area strongly tumid; sides of elytra distinctly margined and almost parallel; apex slightly dehiscent, evenly arcuate laterally and terminating in a pointed, upturned apex (Fig. 88).

Metaventrite and first abdominal ventrite broadly and moderately impressed. First abdominal ventrite with distinct, but poorly defined carinae between metacoxae (less so than in other genera) (Fig. 89); carinae and exceeding hind margin of metacoxal cavities. Apicomedial margin of last ventrite moderately emarginate. Aedeagus distinct, with strongly curled apex; parameres with sparse setae (Fig. 92).

**Female.** Similar to male except last abdominal ventrite is subtruncate and the elytral apices are slightly more produced and elongate than in the male.

##### Intraspecific variation.

This species varies slightly in color from dark brown to black and size, (6.0 mm–6.8 mm).

##### Etymology.

This species is named “*bostrychophallus*” meaning “curly phallus”, which refers to the curled apex of the aedeagus (Fig. 92).

##### Distribution and habitat.

This species has been found in rivers at lower elevations (<500m) throughout the Guiana Shield region in Venezuela, and presumably occurs in northern Brazil and Western Guyana as well (Fig. 1).

##### Associated species.

This species has been found in association with *Lutrochus cauraensis* (Lutrochidae) at Kanarakuni, Venezuela (Maier and Short 2013).

#### Roraima

Kodada & Jäch, 1999

http://species-id.net/wiki/Roraima

Figs 2 , 93, 94, 95

##### Diagnosis.

*Roraima* is a monotypic genus. See species diagnosis.

##### Distribution.

*Roraima* is known only from the type locality on Mount Roraima in Bolívar State, Venezuela (Fig. 2).

##### Habitat.

See species account for habitat information.

**Figures 93–95.**
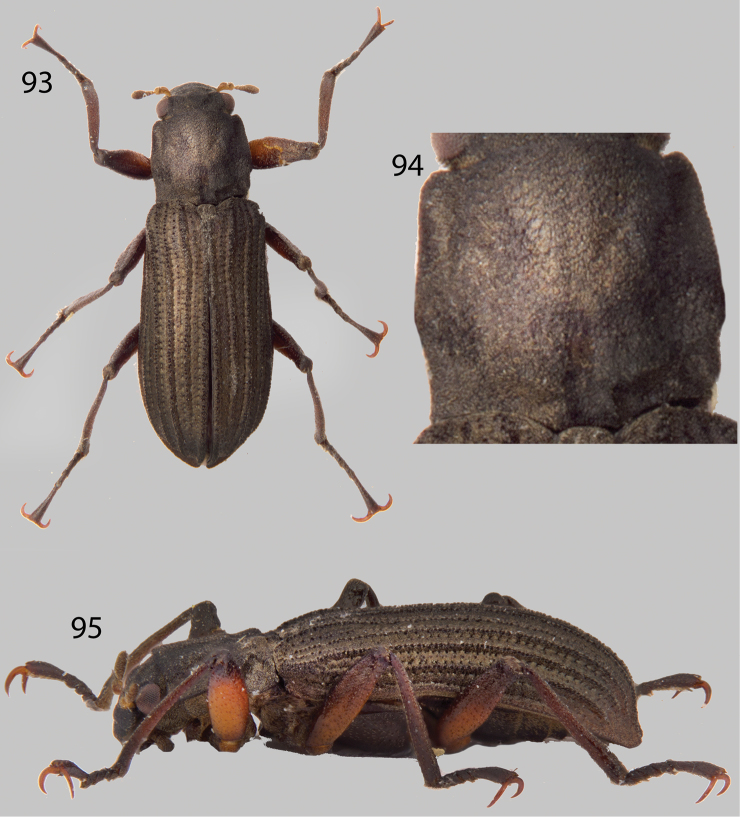
*Roraima carinata*: **93** Dorsal habitus **94** Pronotum, dorsal view **95** Lateral habitus.

#### Roraima carinata

Kodada & Jäch, 1999

http://species-id.net/wiki/Roraima_carinata

Figs 2 , 93, 94, 95

For complete species description and genitalia illustrations see Kodada and Jäch 1999.

##### Material examined.

“SE-Venezuela, Bolívar state, Mt. Roraima; the stream below the waterfall on the south-west face of the Mt. Roraima; which crosses a tourist pathway to Mt. Roraima plateau; ca. 2000 m a.s.l, 3. Feb. 1999” (1 Holotype, NMW).

##### Diagnosis.

This species can be distinguished from all other laraine Elmidae in South America by its large size and distinctive appearance. *Roraima carinata* is the only laraine species larger than 5.0 mm that possesses strongly carinate elytra (Fig. 93) and strongly clubbed antennae. Additionally, the shape of the pronotum is distinct among the Larainae (Fig. 94). The only other species that has carinate elytra is *Hexanchorus leleupi* Delève, and that species is smaller than 5.0 mm and has filiform antennae.

##### Intraspecific variation.

*Roraima carinata* varies in length from 5.1–5.2 mm and slightly in color.

##### Distribution and habitat.

This species is known only from the high elevation type locality. Kodada and Jäch (1999) reported that the aquatic samples which included this species came from a small (ca. 1 m wide) shaded stream with a gravel substrate crossing the tourist path on the southwestern slope of Mount Roraima, in Bolívar State, Venezuela (Fig. 2).

##### Associated species.

The unusual species *Neblinagena doyeli* was found near the *Roraima carinata* collection site, but at a lower elevation.

### Key to the Species of Adult Larainae of Venezuela (Modified from Brown 1981)

**Table:** 

1	Body size smaller, length 2.5 to 5.1 mm	2
1'	Body size larger, length 5.2 to 10.1 mm	16
2 (1)	Ventral surface covered with a thick, silvery mat of setae (Fig. 63); elytra with deep and nearly convergent punctures; antennae clubbed (Fig. 62)	*Phanoceroides* sp. 1
2'	Ventral surface setose (Fig. 19), but never with a thick silvery mat of setae; elytra with fine punctures; antennae variable	3
3 (2')	Pronotum with a deep transverse groove across apical third; body length longer (2.8–5.1 mm) (Fig. 83)	8
3'	Pronotum without a transverse groove, or with a shallow, wide, V-shaped groove across apical third; body length shorter (less than 4.5 mm) (Fig. 74)	4
4 (3')	Pronotum with a shallow, wide, V-shaped groove across apical third (Fig. 53); southern Venezuela *Hypsilara* Maier & Spangler, 2011	5
4'	Pronotum without a transverse groove (Fig. 71). Texas (USA), south to Peru; Greater Antilles; *Phanocerus* Sharp, 1882	6
5 (4)	Elytral apices produced; body size small (TL 3.9–4.2 mm); phallobase ca. 0.6× as long as median lobe; parameres short, ca. 0.67× as long as median lobe	*Hypsilara breweri* Čiampor et al., 2013
5'	Elytral apices rounded; body size large (TL 4.2–4.5 mm); phallobase ca. 0.45× as long as median lobe; parameres long, ca. 0.85x as long as median lobe	*Hypsilara royi* Maier & Spangler, 2011
6 (4')	Pronotum with narrow anterolateral angles, angles not explanate (Fig. 68)	*Phanocerus clavicornis* Sharp, 1882
6'	Pronotum with wide anterolateral angles, angles are explanate (Fig. 71)	7
7 (6')	Body size large, length 3.0–3.5 mm; reddish in color; pronotum 1.6x as wide as long (Fig. 74); mesotibia of male with short setose patch basally (Fig. 75)	*Phanocerus rufus* sp. n.
7'	Body size smaller, length 2.1–2.5 mm; brown in color, pronotum 1.4× as wide as long (Fig. 71); mesotibia of the male with long setose patch basally (Fig. 69)	*Phanocerus congener* Grouvelle, 1898
8 (3)	Pronotum with a median groove and without small prescutellar foveae; anterolateral angles of pronotum rounded (Fig. 83); Costa Rica, south to Venezuela; *Pharceonus* Spangler & Santiago-Fragoso, 1992	9
8'	Pronotum without a median groove and with two small prescutellar foveae; anterolateral angles of pronotum declivous (Fig. 27); Mexico, south to Peru and West Indies; *Hexanchorus* Sharp, 1882	11
9 (8)	Body size small, length less than 3.5 mm; color dark brown (Fig. 85); pronotum with weak bisinuate anterior transverse groove (Fig. 87); male genitalia (aedeagus + phallobase) of medium width (4.3× as long as wide)	*Pharceonus volcanus* Spangler & Santiago-Fragoso, 1992
9'	Body size large, length greater than 3.5 mm; color dark brown to reddish-brown (Fig. 81); pronotum with strong or weak bisinuate anterior transverse groove (Fig. 83); male genitalia variable	10
10 (9')	Color dark brown; male genitalia (aedeagus + phallobase) narrow (6x as long as wide) (Fig. 80); pronotum with only a shallow bisinuate transverse impression across apical third (Fig. 77)	*Pharceonus ariasi* sp. n.
10'	Color reddish-brown; male genitalia (aedeagus + phallobase) wide (3.7× as long as wide) (Fig. 84); pronotum with a deep and strongly bisinuate transverse impression across apical third (Fig. 83)	*Pharceonus grandis* sp. n.
11 (8')	Dorsal habitus with iridescent setae, making the dorsum appear to have a green-gold sheen (Fig. 48)	12
11'	Dorsal habitus lacking iridescent setae (Fig. 37); dorsum setose, but setae lacking iridescent sheen	13
12 (11)	Pronotum with postero-median depression (Fig. 27); mesotibia with short basal pubescent patch, parameres of aedeagus wide	*Hexanchorus dentitibialis* sp. n.
12'	Pronotum without distinct postero-median depression (Fig. 48); metatibia with long basal pubescent patch	*Hexanchorus mcdiarmidi* Spangler & Staines, 2003
13 (11')	Posterior margin of penultimate abdominal ventrite of female with median projection (Fig. 40); aedeagus with parameres long, ca. 0.75–0.80× as long as median lobe (Figure 42)	14
13'	Posterior margin of penultimate abdominal ventrite of female straight, lacking median projection (Fig. 35); aedeagus with parameres short, ca. 0.55–0.65× as long as median lobe (Figure 36)	15
14 (13)	Elytra appearing swollen posteriorly in lateral view (Fig. 45); antennae distinctly filiform (Fig. 44); postero-median impression of pronotum strongly impressed	*Hexanchorus inflatus* sp. n.
14'	Elytra not appearing swollen in lateral view (Fig. 39); antennae serrate or weakly clubbed; postero-median impression of pronotum absent or weakly impressed (Fig. 41)	*Hexanchorus homaeotarsoides* sp. n.
15 (13')	Scutellum convex in lateral aspect; elytral apices of female extended to a long point apically (moderately so in both sexes) (Fig. 28); aedeagus with “can-opener” notch at apex (Fig. 31)	*Hexanchorus falconensis* sp. n.
15'	Scutellum flat in lateral aspect; elytral apices of female not extended to a long point apically (Fig. 35); aedeagus with simple apex (Fig. 36)	*Hexanchorus flintorum* sp. n.
16 (1')	Elytron with distinct longitudinal carinae (Fig. 93). Southern Venezuela	*Roraima carinata* Kodada & Jäch, 1999
16'	Elytron without distinct longitudinal carinae (Fig. 13)	17
17 (16')	Pronotum with a distinct transverse groove across apical third	20
17'	Pronotum without a transverse groove across apical third (Fig. 21). Costa Rica, south to Peru; *Disersus* Sharp, 1882	18
18 (17')	Protibiae of male with dense patch of long, curly setae (Fig. 17)	*Disersus dasycolus* Spangler & Santiago-Fragoso, 1992
18'	Protibiae of male with short, flat setae, similar to setation on entire body (Fig. 10)	19
19 (18')	Metatibiae of male nearly entirely glabrous, sometimes with a small patch of setae basally (Fig. 12)	*Disersus chibcha* Spangler & Santiago, 1987
19'	Metatibiae of male almost entirely pubescent, with a small glabrous patch apically (Fig. 22)	*Disersus inca* Spangler & Santiago-Fragoso, 1992
20 (17)	Pronotum with a lateral longitudinal carina or arcuate-sinuate groove on basal third; Pronotum with two short, converging, prescutellar carinae, each with a deep pit laterally (Fig. 57); *Neblinagena* Spangler, 1985	21
20'	Pronotum without a carina or arcuate-sinuate groove on basal third (Fig. 91)	*Potamophilops bostrychophallus* sp. n.
21 (20)	Pronotum with two prescutellar mammiform tubercles at base and one similar tubercle near each posterolateral angle, thus appearing bidentate (Fig. 61). Venezuela	*Neblinagena prima* Spangler, 1985
21'	Pronotum with two short, converging, prescutellar carinae, each with a deep pit laterally (Fig. 57). Venezuela	*Neblinagena doyeli* Kodada & Jäch, 1999

## Supplementary Material

XML Treatment for Disersus

XML Treatment for Disersus chibcha

XML Treatment for Disersus dasycolus

XML Treatment for Disersus inca

XML Treatment for Hexanchorus

XML Treatment for Hexanchorus dentitibialis

XML Treatment for Hexanchorus falconensis

XML Treatment for Hexanchorus flintorum

XML Treatment for Hexanchorus homaeotarsoides

XML Treatment for Hexanchorus inflatus

XML Treatment for Hexanchorus mcdiarmidi

XML Treatment for Hypsilara

XML Treatment for Hypsilara breweri

XML Treatment for Hypsilara royi

XML Treatment for Neblinagena

XML Treatment for Nebilinagena doylei

XML Treatment for Neblinagena prima

XML Treatment for Phanoceroides

XML Treatment for Phanoceroides

XML Treatment for Phanocerus

XML Treatment for Phanocerus clavicornis

XML Treatment for Phanocerus congener

XML Treatment for Phanocerus rufus

XML Treatment for Pharceonus

XML Treatment for Pharceonus ariasi

XML Treatment for Pharceonus grandis

XML Treatment for Pharceonus volcanus

XML Treatment for Potamophilops

XML Treatment for Potamophilops bostrychophallus

XML Treatment for Roraima

XML Treatment for Roraima carinata
